# Does tai chi improve psychological well-being and quality of life in patients with cardiovascular disease and/or cardiovascular risk factors? A systematic review

**DOI:** 10.1186/s12906-021-03482-0

**Published:** 2022-01-04

**Authors:** Guoyan Yang, Wenyuan Li, Nerida Klupp, Huijuan Cao, Jianping Liu, Alan Bensoussan, Hosen Kiat, Diana Karamacoska, Dennis Chang

**Affiliations:** 1 NICM Health Research Institute, Western Sydney University, Locked Bag 1797, Penrith, NSW 2751 Australia; 2 Hospital of Traditional Chinese Medicine Affiliated to Chengdu University of Traditional Chinese Medicine, Chengdu, 610036 Sichuan China; 3 School of Health Sciences, Western Sydney University, Penrith, NSW 2751 Australia; 4 Center for Evidence-Based Chinese Medicine, Beijing University of Chinese Medicine, Beijing, 100029 China; 5 Faculty of Medicine and Health Sciences, Macquarie University, Sydney, NSW 2109 Australia; 6 Cardiac Health Institute, Sydney, NSW 2122 Australia

**Keywords:** Tai Chi, Stress, Depression, Anxiety, Quality of life, Cardiovascular disease

## Abstract

**Background:**

Psychological risk factors have been recognised as potential, modifiable risk factors in the development and progression of cardiovascular disease (CVD). Tai Chi, a mind-body exercise, has the potential to improve psychological well-being and quality of life. We aim to assess the effects and safety of Tai Chi on psychological well-being and quality of life in people with CVD and/or cardiovascular risk factors.

**Methods:**

We searched for randomised controlled trials evaluating Tai Chi for psychological well-being and quality of life in people with CVD and cardiovascular risk factors, from major English and Chinese databases until 30 July 2021. Two authors independently conducted study selection and data extraction. Methodological quality was evaluated using the Cochrane Risk of Bias tool. Review Manager software was used for meta-analysis.

**Results:**

We included 37 studies (38 reports) involving 3525 participants in this review. The methodological quality of the included studies was generally poor. Positive effects of Tai Chi on stress, self-efficacy, and mood were found in several individual studies. Meta-analyses demonstrated favourable effects of Tai Chi plus usual care in reducing anxiety (SMD − 2.13, 95% confidence interval (CI): − 2.55, − 1.70, 3 studies, *I*^*2*^ = 60%) and depression (SMD -0.86, 95% CI: − 1.35, − 0.37, 6 studies, *I*^*2*^ = 88%), and improving mental health (MD 7.86, 95% CI: 5.20, 10.52, 11 studies, *I*^*2*^ = 71%) and bodily pain (MD 6.76, 95% CI: 4.13, 9.39, 11 studies, *I*^*2*^ = 75%) domains of the 36-Item Short Form Survey (scale from 0 to 100), compared with usual care alone. Tai Chi did not increase adverse events (RR 0.50, 95% CI: 0.21, 1.20, 5 RCTs, *I*^*2*^ = 0%), compared with control group. However, less than 30% of included studies reported safety information.

**Conclusions:**

Tai Chi seems to be beneficial in the management of anxiety, depression, and quality of life, and safe to practice in people with CVD and/or cardiovascular risk factors. Monitoring and reporting of safety information are highly recommended for future research. More well-designed studies are warranted to determine the effects and safety of Tai Chi on psychological well-being and quality of life in this population.

**Systematic review registration:**

International Prospective Register for Systematic Reviews (PROSPERO), CRD42016042905. Registered on 26 August 2016.

**Supplementary Information:**

The online version contains supplementary material available at 10.1186/s12906-021-03482-0.

## Background

### Rationale

Cardiovascular disease (CVD) is the leading cause of death globally. An estimated 17.9 million people died from CVD in 2019, representing 32% of all global deaths [1]. CVDs remain a heavy disease burden on individuals and society. The American Heart Association estimates that the total cost of CVD in the US alone for 2016 was around US$555 billion, and this figure is expected to increase significantly to $1.1 trillion by 2035 [2].

Regular physical activity has been recommended in interventional guidelines for cardiac event prevention and risk factor management [3, 4]. Exercise-based cardiac rehabilitation (CR) is effective in supporting long-term lifestyle changes, reducing the risk of cardiovascular mortality, improving health-related quality of life (QoL), and reducing hospital readmissions [5, 6]. Given the benefits, the uptake of CR remains suboptimal. Surveys in the United States, Australia, and Europe have demonstrated that less than 20% of eligible patients are receiving CR [7–9]. Of note, the majority of CR programs continue to offer the traditional models of care employed since the 1980s and 90s [10]. Furthermore, although stress, anxiety, and depression have been increasingly recognised as the potential, modifiable non-physical cardiovascular risk factors that are associated with the development of CVD [11, 12], they are frequently undertreated among people with CVD [13] or cardiovascular risk factors [14]. Given the number of people with CVD is increasing worldwide [1], a novel, effective and alternative exercise option to increase the uptake of CR and improve psychological outcomes and QoL in these populations is needed [15, 16].

Tai Chi has the potential to be such as adjunct intervention option. As a traditional mind-body exercise, it contains the Chinese folk and military martial arts, breathing and meditative techniques, philosophy of *yin* and *yang*, and traditional Chinese medicine theories [17]. In the past decades, the clinically important benefits of Tai Chi has been demonstrated in previous meta-analyses of randomised controlled trials for improving cardiac output, cardiorespiratory endurance, and reducing blood pressure, blood lipid profiles, and blood glucose in people with coronary heart disease, chronic heart failure, hypertension, hyperlipidaemia, and type 2 diabetes [18–20].

The benefits of Tai Chi for psychological well-being and quality of life (QoL) in people with CVD and cardiovascular risk factors, however, have not yet been determined. Although several previous systematic reviews [21–25] have reported the beneficial effects of Tai Chi on several psychological outcomes, these studies enrolled a combination of various populations rather than people with CVD. One meta-analysis [26] found significant effects of Tai Chi-based CR in improving QoL and lowering anxiety and depression in people with coronary heart disease, but the benefits of Tai Chi for other CVDs and risk factors were not evaluated in this review. One recent meta-analysis involving 15 English or German-language studies found that Tai Chi significantly improved QoL and reduced depression and psychological distress in people with CVDs [27]. Of note, the majority of clinical studies on Tai Chi were published in the Chinese language [28, 29], which have not yet been critically evaluated and reported in English. Hence, a comprehensive and critical review is needed to determine the effect of Tai Chi in this area.

### Objectives

We aimed to evaluate the effects and safety of Tai Chi on psychological well-being and QoL in people with CVD and/or cardiovascular risk factors.

## Methods

### Protocol and registration

The protocol of this systematic review was registered in the International Prospective Register for Systematic Reviews (PROSPERO) (ID: CRD42016042905) and published in *BMJ Open* [30].

### Literature search

We searched for relevant randomised control trials (RCTs) regardless of their publication status (e.g., published, unpublished, in press, or preprint). The search terms in English databases were “Tai Chi”, “Tai Chi Chuan”, “Tai Chi Chih”, “ta’i chi”, “Tai Ji Quan”, “taijiquan”, “cardiovascular disease”, “coronary heart disease”, “stroke”, “heart failure”, “hypertension”, “high blood pressure”, “diabetes”, “dyslipidaemia”, “high cholesterol”, “randomised controlled trial”, “randomized controlled trial”, “controlled clinical trial”, “randomly”, “clinical”, “trial”, “random”, “randomised” and “randomized”. The search terms in Chinese databases were “*Tai_ji* (Tai Chi)”, “*Tai_ji_chuan* (Tai Chi)”, “Xin_xue_guan_bing (cardiovascular disease)”, “Gao_xue_ya (hypertension)”, “Tang_niao_bing (diabetes)”, “Gao_xue_zhi (dyslipidaemia)” and “*sui_ji* (randomized)”. Table S1 presents two examples of the search syntaxes used to search English and Chinese databases.

We conducted electronic searches in the following major English and Chinese databases from their inception to 30 June 2019 and an update search till 30 July 2021: Cochrane Library, EMBASE (from 1947), PubMed (from 1966), Sino-Med database (from 1978), China National Knowledge Infrastructure (CNKI, from 1979), VIP Journal Integration Platform (VJIP, from 1989), and Wanfang Data Chinese database (from 1985).

We also searched the following trials registers to identify any unpublished data from completed trials: Australian New Zealand Clinical Trials Registry (ANZCTR) (www.anzctr.org.au), Chinese Clinical Trial Registry (ChiCTR) (www.chictr.org.cn/enindex.aspx), Clinical Research Information Service (CRiS) (https://cris.nih.go.kr/), Current Controlled Trials (www.controlled-trials.com), and ClinicalTrials.gov (www.clinicaltrials.gov). The search was also conducted till 30 June 2019 and an update search till 30 July 2021. Additional clinical trials were also identified by manually searching the reference lists of relevant studies.

### Eligibility criteria

#### Type of study

We included parallel RCTs or the first phase data of randomised cross-over clinical trials.

#### Type of participants

We included participants aged 40 years or older regardless of gender with a diagnosis of CVD including myocardial infarction, coronary heart disease (CHD), heart failure and stroke, or with cardiovascular risk factors including hypertension, diabetes, and/or high blood lipids.

#### Type of intervention

All types of Tai Chi were eligible for inclusion, regardless of the forms (such as 24-form, 54-form, and 83-form Tai Chi), or styles (such as *Chen*, *Yang*, *Wu,* and *Sun* style).

#### Type of control

No treatment, waitlist, sham control, other forms of exercise, and conventional treatments were eligible for inclusion. Comparisons also included a co-intervention if applied in all arms.

#### Type of outcome

The primary outcome was psychological stress measured by validated instruments. The secondary outcomes were other psychological measures including anxiety, depression, mood disturbance, self-esteem and QoL, and adverse events.

### Study selection

Two authors (GYY and WYL) independently screened the titles and abstracts. Full-text reports of potentially relevant studies were retrieved and examined against the eligibility criteria. We contacted study authors by email to obtain key unpublished information that is missing from reports of included studies, including details to inform risk of bias assessments, interventions, outcomes, and results.

### Data collection and data items

Two authors (GYY and WYL) independently extracted data from the included trials using a pre-defined data extraction form. Any disagreement about the data extraction was resolved by discussion, and another author arbitrated when necessary (NK).

The extracted data included: (1) publication information: authors, country, and year of publication; (2) study design: method of random number generation, allocation concealment, and blinding; (3) participants: sample size and characteristics of participants (i.e. age, gender, duration and severity of the disorder); (4) interventions: type and/or form of Tai Chi, details of treatment and control; and (5) outcome data: outcome measures and main findings after treatment and at follow-up periods if available. To deal with missing data or unclear information, we contacted the original authors by email to clarify.

### Risk of Bias assessment

We used the risk of bias tool recommended by the Cochrane Collaboration [31]. The following items were assessed for each study: selection bias (random sequence generation and allocation concealment); detection bias (blinding of outcome assessment); attrition bias (incomplete outcome data); reporting bias (selective reporting); and other bias. We did not rate the performance bias, considering the difficulty to blind the participants and personnel in Tai Chi studies. For each item, there are three potential bias judgements: ‘low risk’, ‘high risk’, or ‘unclear risk’. A clinical trial that met the criteria was judged as having a low risk of bias, a trial that did not meet the criteria was judged as having a high risk of bias, and a trial with insufficient information to judge was classified as unclear risk of bias. Any disagreements were resolved by discussion, with the involvement of a third author (NK) where necessary.

### Grading the quality of evidence

We applied the Grading of Recommendations Assessment, Development and Evaluation (GRADE) approach for rating the quality of evidence [32]. A body of evidence based on RCTs begins as high-quality evidence, and we took the following factors into account when deciding whether or not to downgrade the quality of evidence for each outcome: study limitations, inconsistency of results, imprecision, indirectness of evidence, and reporting bias [32]. The GRADE classifies the quality of evidence into four levels:

- High quality: further research is very unlikely to change our confidence in the estimate of effect.
- Moderate quality: further research is likely to have an important impact on our confidence in the estimate of effect and may change the estimate.
- Low quality: further research is very likely to have an important impact on our confidence in the estimate of effect and is likely to change the estimate.
- Very low quality: any estimate of effect is very uncertain [32].

We presented the evidence for main outcomes in ‘Summary of findings’ (SoF) tables using the GRADEPro web application.

### Synthesis of results

We summarised data using mean difference (MD) with 95% confidence interval (CI) for continuous outcomes. We used standardised mean difference (SMD) as a summary statistic in the meta-analysis when the included studies assessed the same outcome using various instruments [33]. Clinical heterogeneity was assessed according to the characteristics of the included studies and participants, and details of the interventions and measurements. We assessed statistical heterogeneity by the *I*^2^ statistic. The statistical heterogeneity was regarded as substantial if the *I*^*2*^ statistic was greater than 50%, and as considerable if the *I*^*2*^ statistic was greater than 75% [31, 34].

We performed statistical analyses with Cochrane’s Review Manager software (version 5.3). We pooled data if the *I*^2^ statistic was less than 75%. We used the random-effects model to conduct the meta-analysis unless the *I*^2^ statistic was less than 25%. Subgroup analyses were conducted to find the cause and explain the heterogeneity. Funnel plots were performed to detect publication bias when more than 10 trials were included in the meta-analysis. Post-hoc subgroup analyses were conducted for CVD risk factors (i.e., hypertension and type 2 diabetes mellitus (T2DM)), and each included CVD (i.e., CHD, Stroke, and HF).

## Results

### Studies selection

**Fig. 1:**
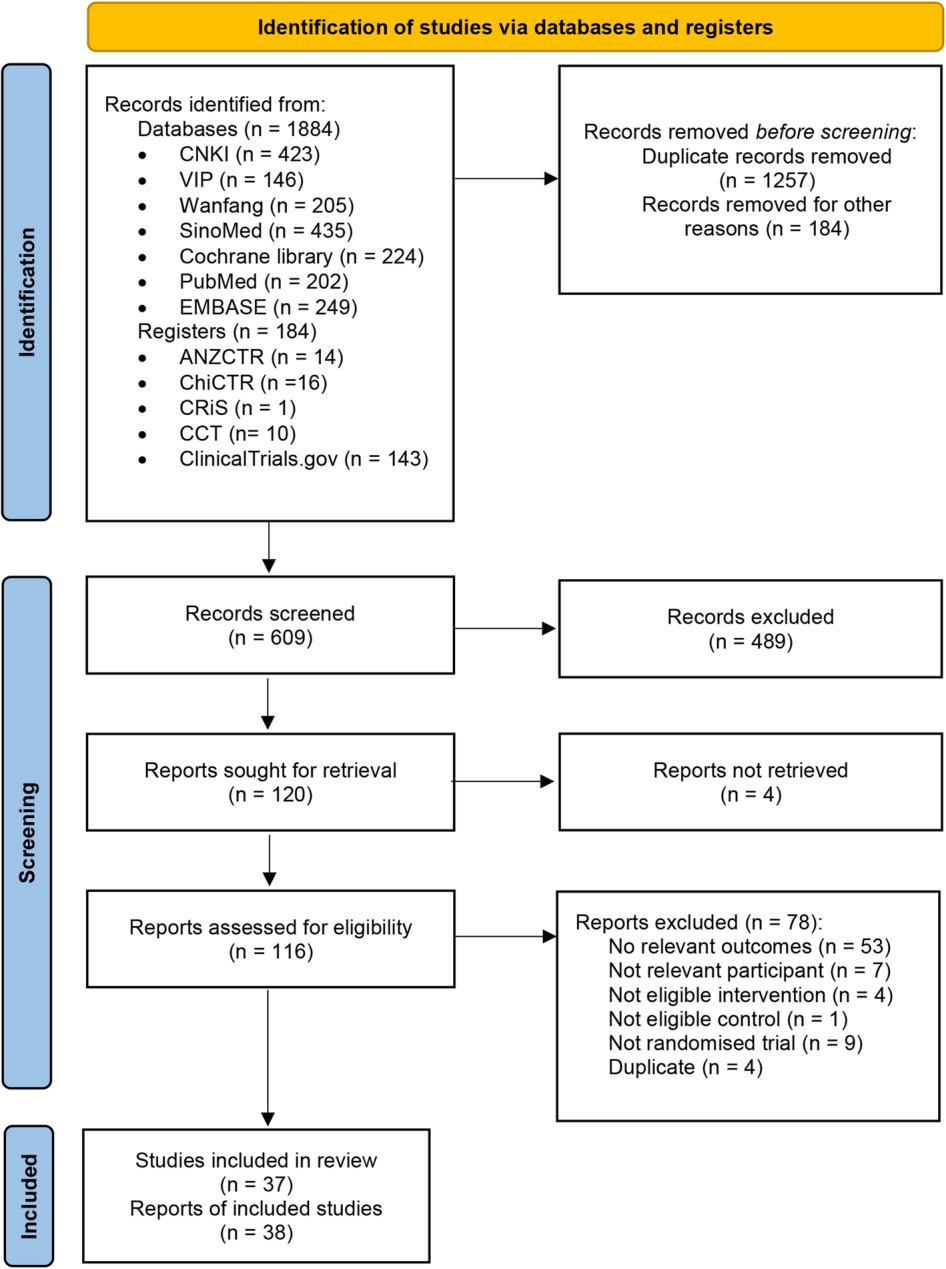
PRISMA Flow Diagram. Note: PRISMA, Preferred Reporting Items for Systematic Reviews and Meta-Analyses: The PRISMA Statement, which is used worldwide to improve the reporting of systematic reviews and meta-analyses

We initially identified 1884 studies, from which 1257 were excluded as duplicates. We screened the remaining 609 studies by titles and abstracts, and 120 full-text articles were retrieved to identify eligibility. Four studies were not retrieved, and 78 studies were excluded for specific reasons. Finally, a total of 37 studies (38 reports) met the inclusion criteria [35–72]. The selection procedure is shown in a PRISMA Flow Diagram (Fig. 1).

### Studies characteristics

Of the included reports, 16 were published in English and 22 in the Chinese language between 2004 and 2021. These studies were conducted in China, the United Kingdom, the United States, Italy, South Korea, and Australia. There were 12 studies that enrolled people with chronic heart failure [35, 36, 38, 45, 49–51, 62, 64–67, 72], nine studies with type 2 diabetes [41, 48, 52, 57–59, 63, 68, 69], eight studies with coronary heart diseases [39, 40, 43, 44, 47, 61, 70, 71], seven studies with hypertension [37, 42, 46, 53, 55, 56, 60], and one study with stroke [54]. The sample size of the included studies ranged from 16 to 326, with an average of 95, totalling 3525 participants. Table 1 shows the characteristics of the included studies.

**Table 1:** Characteristics of included studies on Tai Chi for CVD and/or risk factors

Study ID	Disease/ condition	Sample size	Intervention	Duration (weeks)	Control	Lost to follow up (No. (%))	Outcome measures
Barrow DE 2007 [35]	Symptomatic heart failure	65	Wu Chian Chuan style Tai Chi, twice 55-min sessions weekly + Usual medical care	16	Usual medical care	13/65 (20%) in total; I: 7/32 (21.8%); C: 6/33 (18.1%)	Safety; mood (SCL-R); QoL (MLHF)
Caminiti G 2011 [36]	Chronic heart failure	60	A modified 10-form Yang-style Tai Chi, three 50-min sessions weekly + Endurance Training + Usual medical care	12	Endurance Training: three 50-min sessions weekly + Usual medical care	3/60 (5.0%) in total; I: 0; C: 3/30 (10.0%)	Safety; QoL (MacNewQLMI)
Chan AWK 2018 [37]	Hypertension	246	24 simplified Tai Chi, 60 min per session, 2 sessions weekly	12	Control 1: Aerobic exercise Control 2: No treatment	28/246 (11.4%) in total; I: 13/82 (15.9%); C1: 19/82 (23.2%); C2: 20/82 (24.4%)	Stress (PSS-10), Exercise self-efficacy (TCSE&SEE), QoL (SF-12)
Cui H 2020 [38]	Chronic heart disease	44	Tai Chi + conventional pharmacologic therapy	12	Aerobic exercise + conventional pharmacologic therapy	2/44 (4.5%) in total; C: 2/22 (9.1%)	Safety; QoL (MLHF)
Ding FM 2013 [39]	Acute myocardial infarction after PCI	90	42-form Chen style Tai Chi, at least five 60-min sessions per week + Behaviour guidance + Usual medical care + Jogging	24	Control 1: Behaviour guidance + Usual medical care Control 2: Jogging	NR	QoL (SF-36)
Fan QY 2020 [40]	Coronary heart disease	86	Tai Chi + usual treatment and care	NA	Usual treatment and care (medication, psychological, diet, walking)	NA	QoL (SF-36)
Gong ZY 2020 [41]	T2DM	20	Tai Chi (eight fundamental movements and five steps)	12	C1: usual medication treatment C2: Tai Chi + sand table game	NA	Depression (SDS), anxiety (SAS)
Han QY 2010 [42]	Hypertension	60	24 simplified Yang-style Tai Chi, 45-60 min per session, 1-2 sessions daily + Usual medical care	240	Usual medical care	2/60 (3.3%) in total; I: 0; C: 2/30 (6.6%)	Safety, QoL (SF-36)
Li Y 2019 [43]	Chronic heart failure	326	Five movements and 24-form Yang style Tai Chi, 1 h per session daily + conventional treatment and care (the treatment of CHD symptoms, appropriate diets, exercises, medicine, and psychological therapy)	24	Conventional treatment and care (the treatment of CHD symptoms, appropriate diets, exercises, medicine, and psychological therapy)	77/326 (23.6%) in total: I: 35/163 (21.5%); C: 42/163 (25.8%)	QoL (SF-36), Depression (SDS), anxiety (SAS)
Liu J 2020 [44]	Chronic heart failure	70	24-form Tai Chi, 50-60 min per session, twice a day + same treatment as control	40	Routine treatment, examination, nursing, and health education. Antidepressant amitriptyline was administered at a dose of 50–200 mg/day according to the different severity degrees of depression	9/70 (12.9%) in total: I: 5/35 (14.3%); C: 4/35 (11.4%)	QoL (SF-36), Depression (SDS), anxiety (SAS)
Luberto CM 2020 [45]	Heart failure	100	5 simplified Yang-style Tai Chi, twice 60-min sessions weekly and home practice > 3 times weekly + Usual medical care + General exercise advice	12	Usual medical care + General exercise advice + Health education	4/100 (4.0%) in total; I: 1/50 (2.0%); C: 3/50 (6.0%)	Depression (EPOMS), QoL (MLFHQ), Social support (The Multidimensional Scale of Perceived Social Support), Cardiac exercise self-efficacy (a 16-item measure used to assess an individual’s self-efficacy for exercise-related activities)
Ma CH 2018 [46]	Hypertension	158	24 simplified Tai Chi, two 90-min sessions weekly & home practice in group + Usual medical care	29	Usual medical care	45/158 (28.5%) in total; I:24/79 (30.4%); C:21/79 (26.6%)	Social support (SSRS), Depression (CES-D), QoL (SF36)
Ma CJ 2020 [47]	Coronary heart disease	32	Tai Chi (24-form simplified Tai Chi) + usual medication treatment	12	Usual medication treatment	2/32 (6.25%) in total;	Safety; QoL (SF-36)
Meng E 2014 [48]	Type 2 diabetes	200	Tai Chi + Health education + Diet guidance + Usual medical care	12	Health education + Dietary guidance + Usual medical care	NR	QoL (SF-36)
Pan XF 2016 [49]	Chronic heart failure	61	24 simplified Yang-style Tai Chi, one 30-min session daily + Health education + Diet guidance + Usual medical care	24	Health education + Diet guidance + Usual medical care	NR	QoL (SF-36)
Redwine LS 2019 [50]	Heart failure	70	Yang-style Tai Chi Chuan-Short Form (first third), twice 60-min sessions weekly and practice at home for 10-20 min/day, on non-class days + usual care (including regular visits to their cardiologist, primary care physicians, and other health specialists	16	resistance band (RB) (based on the Center for Disease Control’s “Move” program), twice 60-min sessions weekly and practice at home for 10-20 min/day, on non-class days + usual care (including regular visits to their cardiologist, primary care physicians, and other health specialists / usual care (including regular visits to their cardiologist, primary care physicians, and other health specialists	11/70 (15.7%) in total; I: 4/25 (16.0%); C1: 3/22 (13.6%); C2: 4/23 (17.4%)	Depression (BDI)
Sang L 2015 [51]	Chronic heart failure	100	Specially designed Tai Chi program, one 15-min session daily + Usual medical care	12	Usual medical care	NR	QoL (MLHF)
Shen XY 2019 [52]	T2DM	108	Tai Chi + usual medication treatment	12	Usual medication treatment + walking	7/108 (6.5%) in total; I:2/54 (3.7%); C: 5/54 (9.3%)	QoL (DAQL), Depression (GDS)
Shou XL 2019 [53]	Hypertension	208	24-Style Simplified Tai Chi + same general daily lifestyle intervention as control	12	General daily lifestyle advice (hypertension knowledge propaganda, propaganda for blood pressure monitoring, and healthy lifestyle self-management, such as persuasion for smoking cessation, alcohol restriction, sodium restriction, dietary balance, weight control, and general daily exercise)	10/208 (4.8%) in total; I:6/104 (5.8%); C: 4/104 (3.8%)	QoL (SF-36)
Song R 2021 [54]	Stroke	34	Tai Chi-based stroke rehabilitation program	24	Stroke-specific symptom management program	5/34 (14.7%) in total; I:3/18 (16.7%); C: 4/104 (12.5%)	QoL (SS-QOL)
Sun F 2014 [55]	Hypertension	90	24 simplified Yang style Tai Chi, one 2-h session daily	8	Health education	10/90 (11.1%) in total; I: 7/45 (15.5%); C: 3/45 (6.6%)	Depression (SDS), anxiety (SAS)
Sun J 2015 [56]	Hypertension	300	Tai Chi in group 3 h & 2 h home practice weekly	48	Active controls: non-exercise-related activities such as reading	35/300 (11.6%) in total; I: 14/150 (9.3%); C: 20/150 (13.3%)	QoL (SF-12)
Tsang T 2007 [57]	Type 2 diabetes	38	Tai Chi for Diabetes (a 12-movement hybrid from Sun and Yang styles), twice 1-h sessions weekly	16	Sham exercise (e.g., seated calisthenics & gentle stretching)	1/38 (2.6%) in total; I: 1/18 (5.5%); C: 0	Safety; QoL (SF-36)
Wang HP 2014 [58]	Type 2 diabetes	70	24 simplified Yang-style Tai Chi, five 40-min sessions weekly + Diet guidance + Usual medical care	8	Dietary guidance + Usual medical care	NR	Mood (SCL-90)
Wang P 2009 [59]	Type 2 diabetes	64	24 simplified Yang-style Tai Chi, 45-60 min per session, 5-7 sessions weekly + Health education + Usual medical care	24	Health education + Usual medical care	0	QoL (SF-36)
Wang XB 2019 [60]	Hypertension	100	Tai Chi (24-form simplified Tai Chi) + usual treatment and care	12	Usual treatment and care (medication, daily life behaviour, psychological, diet, exercise)	NA	QoL (WHO-BREF), anxiety (SAS)
Wang XK 2013 [61]	Acute myocardial infarction after PCI	60	42-form Chen style Tai Chi, five 60-min sessions weekly + Behaviour guidance + Usual medical care	24	Behaviour guidance + Usual medical care	NR	QoL (SF-36)
Wang YH 2019 [62]	Chronic heart failure	50	Tai Chi + usual medication treatment	8	C1: usual treatment (medication, health education, diet guidance) C2: usual medication + Tai Chi + external counterpulsation	NA	QoL (SF-36)
Wu F 2010 [63]	Type 2 diabetes	40	24 simplified Yang-style Tai Chi, 60-min per session, > 3 sessions weekly + Usual medical care	24	Usual medical care	NR	QoL (SF-36)
Yao CD 2010 [64]	Chronic heart failure	150	42-form Chen style Tai Chi, 5-15 min per session (30 min per session after the first month), > 5 sessions weekly + Lifestyle guidance + Usual medical care	24	Lifestyle guidance + Usual medical care	NR	QoL (MLHF)
Yeh GY 2004 [66]	Chronic heart failure	30	5-form simplified Yang-style Tai Chi, twice 60-min sessions weekly & home practice > 3 times weekly + Usual medical care + Dietary guidance + General exercise advice	12	Usual medical care + Dietary guidance + General exercise advice	0	Safety; QoL (MLHF)
Yeh GY 2011 [65]	Chronic heart failure	100	5-form simplified Yang-style Tai Chi, twice 60-min sessions weekly & home practice > 3 times weekly + Usual medical care + General exercise advice	12	Usual medical care + General exercise advice + Health education	4/100 (4.0%) in total; I: 1/50 (2.0%); C: 3/50 (6.0%)	Safety; mood (POMS), psychosocial functioning (CESI); QoL (MLHF)
Yeh GY 2013 [67]	Heart failure with a preserved ejection fraction	16	5-form simplified Yang-style Tai Chi, twice 60-min sessions weekly & home practice > 3 times weekly + Usual medical care + General exercise advice	12	Usual medical care + General exercise advice + Aerobic exercise, twice 1-h weekly	0	Safety; mood (POMS), self-efficacy (SEBES); QoL (MLHF)
Yin NN 2020 [68]	T2DM	68	Tai Chi (18-form Chen-style)	12	C1: Healthy Qi Gong; C2: Health education (knowledge about diabetes, management interventions, nursing for complications, and healthy lifestyle behaviours)	15/68 (22.1%) in total; I:9/33 (27.3%); C: 6/35 (17.1%)	Well-being (Index of Well-Being), depression (CES-D)
Zhang EM 2014 [69]	Type 2 diabetes with depression (SDS > 40)	40	24 simplified Yang-style Tai Chi, 60-min per session + Usual medical care	14	Usual medical care + Walking (80-100 steps/min)	NR	Depression (SDS)
Zhang GW 2020 [70]	Coronary heart disease	36	Tai Chi + Traditional Chinese medicine (including Danshen and Suxiao Jiuxin Pills) + health education lesson	12	Usual lifestyle + equal amount of physical activities + Traditional Chinese medicine (including Danshen and Suxiao Jiuxin Pills) + health education lesson	6/36 (16.7%) in total; I:1/19 (5.3%); C: 5/17 (29.4%)	QoL (CQQC)
Zhang SQ 2011 [71]	Acute myocardial infarction after PCI	132	42-form Chen style Tai Chi, 5-15 min per session (30-min session after the first month), > 5 sessions weekly + Behaviour guidance + Usual medical care	48	Behaviour guidance + Usual medical care	NR	QoL (MLHF)
Zhou B 2020 [72]	Heart failure	103	Tai Chi + Cardiac rehab	12	Cardiac rehab (heath education, usual medication, diet guidance, exercise)	NA	Depression (HAMD, SDS), QoL (MLHF)

*Abbreviations*: *PCI* Percutaneous coronary intervention, *QoL* Quality of life, *STAI* State and anxiety inventory, *PSS* The Perceived Stress Scale, *CES-D* The Center for Epidemiological Studies-Depression, *MOS* Medical Outcomes Study, *SSRS* Social Supporting Rating Scale, *SPS* The revised Social Provision Scale, *MAAS* The Mindful Attention Awareness Scale, *SCS-R* The revised Self-Compassion Scale, *SIBS-R* The revised Spiritual Involvement and Beliefs Scale, *MLHFQ* The Minnesota Living with Heart Failure questionnaire, *WHOQOL-100* The World Health Organization Quality of Life, *SAS Zung* Self-Rating Anxiety Scale, *SCL-90* Symptom Checklist-90, *SCL-R* Symptom Checklist-90-Revised, *POMS* The Profile of Mood States, *SEBES* The Self-Efficacy-Barriers to Exercise Scale

Various Tai Chi styles and forms, time per session, frequencies, and durations were utilised. The majority of studies applied the *Yang* style, and the most popular one was the 24-form Simplified *Yang* style. Most studies practiced a modified/simplified version of Tai Chi in group classes, under the supervision and instruction of a professional Tai Chi instructor, an experienced trainer, or exercise physiologist. Home practice, in addition to group classes, was encouraged or required in nine studies [35, 37, 38, 50, 54, 56, 65–67]. The frequency and duration varied from 5 to 120 min (standardised or increased gradually) per session, 2 to 14 sessions per week, lasting 8 to 240 weeks. Table S2 lists the details of the Tai Chi interventions used in the included studies.

### Methodological quality

We contacted the corresponding authors by email to clarify unclear or missing information in the papers; however, the response rate was low. Accordingly, only three studies were rated as low risk of bias in all six items, and the majority were rated as unclear risk of bias. The overall methodological quality of the included studies was poor (Fig. S1 and S2).

Although all included studies mentioned ‘randomised’, only 21 studies (21/37, 56.8%) reported the methods for sequence generation, including ‘random number table’ and ‘computer-generated allocation method’. Seven studies (7/37, 18.9%) reported information on allocation concealment, eight studies (8/37, 21.6%) reported the blinding of outcome assessors, 21 studies (21/37, 55.6%) reported information about participants lost to follow up, and 10 studies (10/37, 27.0%) reported their protocol registration.

### Effects of interventions

The effect estimates of Tai Chi for psychological well-being and QoL in people with or at risk of CVD and the post-hoc subgroup analyses were shown in Table S3 and Table S4, respectively. Tables 2 and 3 respectively present the summary of main findings when Tai Chi plus usual care was compared with usual care alone and Tai Chi was compared with aerobic exercise. Table S5 presents the GRADE certainty assessment in detail.

**Table 2:** Summary of findings: Tai Chi plus usual care compared to usual care for psychological well-being and QoL in people with CVD and risk factors

Outcomes	Anticipated absolute effects^*^ (95% CI)		Relative effect (95% CI)	№ of participants (studies)	Certainty of the evidence (GRADE)
	Risk with Usual care	Risk with Tai Chi + Usual care			
Safety	16 per 1000	**8 per 1000** (3 to 20)	**RR 0.50** (0.21 to 1.20)	248 (5 RCTs)	⨁◯◯◯ VERY LOW ^a, b^
Stress assessed with: PSS-14	The mean stress was **3.99** scores	MD **0.76 scores lower** (1.02 lower to 0.5 lower)	–	61 (1 RCT)	⨁◯◯◯ VERY LOW ^c, d^
Anxiety assessed with: HADS-A & SAS	–	SMD **2.13 lower** (2.55 lower to 1.7 lower)	–	410 (3 RCTs)	⨁⨁◯◯ LOW ^c^
Depression assessed with: HADS-D, GDS, BDI, SDS & CES-D	–	SMD **0.85 SD lower** (1.52 lower to 0.17 lower)	–	675 (6 RCTs)	⨁⨁◯◯ LOW ^a, e^
Quality of Life - Mental Health assessed with: SF-36	The mean quality of Life - Mental Health was **48.1 - 87.01** scores	MD **7.86 scores higher** (5.2 higher to 10.52 higher)	–	1124 (11 RCTs)	⨁⨁◯◯ LOW ^c^
Quality of Life assessed with: Total score of SF-36	The mean quality of Life was **61.5 - 82.96** scores	MD **18.91 scores higher** (12.8 higher to 25.03 higher)	–	369 (3 RCTs)	⨁⨁◯◯ LOW ^c^

*The risk in the intervention group (and its 95% confidence interval) is based on the assumed risk in the comparison group and the relative effect of the intervention (and its 95% CI)

*CI* Confidence interval, *RR* Risk ratio, *MD* Mean difference, *SMD* Standardised mean difference

GRADE Working Group grades of evidence High certainty: We are very confident that the true effect lies close to that of the estimate of the effect Moderate certainty: We are moderately confident in the effect estimate: The true effect is likely to be close to the estimate of the effect, but there is a possibility that it is substantially different Low certainty: Our confidence in the effect estimate is limited: The true effect may be substantially different from the estimate of the effect Very low certainty: We have very little confidence in the effect estimate: The true effect is likely to be substantially different from the estimate of effect

Explanations:

a. Moderate risk of bias (RoB), and no sensitivity analysis of only low RoB studies was conducted or if conducted, the effect estimates were unstable

b. Optimal information size (OIS) is not met, 95% CI overlaps no effect but fails to include both important benefit and harm

c. High RoB, and no sensitivity analysis by excluding high RoB studies was conducted or if conducted, the effect estimates were unstable

d. OIS is not met, 95% CI excludes overlap no effect

e. *I*^*2*^ > 75% & all studies favour one direction (visual inspection)

**Table 3:** Summary of findings: Tai Chi compared to aerobic exercise for psychological well-being and QoL in people with CVD and risk factors

Outcomes	Anticipated absolute effects^*^ (95% CI)		Relative effect (95% CI)	№ of participants (studies)	Certainty of the evidence GRADE)
	Risk with Aerobic exercise	Risk with Tai Chi			
Safety	150 per 1000	**182 per 1000** (47 to 715)	**RR 1.21** (0.31 to 4.77)	42 (1 RCT)	⨁◯◯◯ VERY LOW ^a, b^
Stress assessed with: PSS-10	The mean stress was **10.28** scores	MD **2.09 scores lower** (4.22 lower to 0.04 higher)	–	132 (1 RCT)	⨁⨁◯◯ LOW ^c^
Depression assessed with: SDS & POMS	–	SMD **0.1 SD lower** (0.62 lower to 0.43 higher)	–	56 (2 RCTs)	⨁◯◯◯ VERY LOW ^a, b^
Quality of life measured by MLHF	The mean quality of life measured by MLHF was **25.6 - 28.7** scores	MD **1.55 scores higher** (8.5 lower to 11.59 higher)	–	58 (2 RCTs)	⨁◯◯◯ VERY LOW ^a, b^

*The risk in the intervention group (and its 95% confidence interval) is based on the assumed risk in the comparison group and the relative effect of the intervention (and its 95% CI)

*CI* Confidence interval, *RR* Risk ratio, *MD* Mean difference, *SMD* Standardised mean difference

GRADE Working Group grades of evidence

High certainty: We are very confident that the true effect lies close to that of the estimate of the effect

Moderate certainty: We are moderately confident in the effect estimate: The true effect is likely to be close to the estimate of the effect, but there is a possibility that it is substantially different

Low certainty: Our confidence in the effect estimate is limited: The true effect may be substantially different from the estimate of the effect

Very low certainty: We have very little confidence in the effect estimate: The true effect is likely to be substantially different from the estimate of effect

Explanations:

^a^Moderate risk of bias (RoB), and no sensitivity analysis of only low RoB studies was conducted or if conducted, the effect estimates were unstable

^b^Optimal information size (OIS) is not met, 95% CI overlaps no effect, and both important benefit and harm included (i.e., very wide CI)

^c^OIS is not met, 95%CI overlaps no effect but fails to include both important benefit and harm

### Stress

#### Narrative synthesis

Two studies [37, 44] reported the effects of Tai Chi on stress. We did not pool their data because of the different comparisons applied in their studies.

Liu et al. (2020) [44] compared Tai Chi combined with usual care and usual care alone in people with CHD, and measured stress with the Perceived Stress Scale 14-item (PSS-14) (Range from 0 to 56; a higher score indicates greater stress). A significantly greater stress reduction was found in the Tai Chi plus usual care group, compared with usual care alone (including usual pharmacologic therapy, examination, nursing, and health education) (Very low certainty) (Table 2, Table S5).

Chan et al. (2018) [37] compared Tai Chi with aerobic exercise alone or no treatment in people with hypertension, and measured stress with the Perceived Stress Scale 10-item (PSS-10) (Range from 0 to 40; higher scores reflect higher levels of perceived stress). This study showed that the Tai Chi group achieved a significantly greater reduction in stress than the control and aerobic exercise groups (Low certainty) (Table 3, Table S5).

### Anxiety

Five studies [41, 43, 44, 55, 60] investigated the effects of Tai Chi on anxiety.

#### Meta-analysis

The meta-analysis indicated that Tai Chi in combination with usual care is superior in reducing anxiety (SMD -2.13, 95% CI: − 2.55, − 1.70, 3 studies, *I*^*2*^ = 60%) (Low certainty) to usual care alone (including pharmacologic therapy, advice on medication, daily life behaviour, psychological support, diet, and exercise), in people with CHD or hypertension (Table 2, Table S5). A subgroup analysis found similar results among people with CHD (SMD -1.98, 95% CI: − 2.65, − 1.31, 2 studies, *I*^*2*^ = 76%) (Table S4).

#### Narrative synthesis

Two studies [41, 55] reported anxiety as measured by *Zung* Self-Rating Anxiety Scale (SAS) (scale from 20 to 80; a lower score indicates less anxiety), and found that the Tai Chi group experienced a significantly greater reduction in anxiety than the usual pharmacological therapy intervention for people with T2DM [41], and health education in people with hypertension [55].

### Depression

Eleven studies [41, 43–46, 50, 52, 55, 68, 69, 72] reported the effects of Tai Chi on depression.

#### Meta-analysis

Findings from the meta-analysis indicated that Tai Chi in combination with usual care significantly improved depression (SMD -0.86, 95% CI: − 1.35, − 0.37, 6 studies, *I*^*2*^ = 88%) (Low certainty), compared with usual care alone, in people with CHD, hypertension, or T2DM (Table 2, Table S5). Subgroup analyses for CHD, HF, and T2DM demonstrated consistent findings, but could not identify the source of the statistical heterogeneity in the meta-analysis (Table S4).

Another two meta-analyses found that Tai Chi was equally effective in reducing depression compared with that of aerobic exercise (SMD -0.10, 95% CI: − 0.62, 0.43, 2 studies, *I*^*2*^ = 0%) (Very low certainty) (Table 3, Table S5) or health education (SMD -0.11, 95% CI: − 0.78, 0.56, 2 studies, *I*^*2*^ = 76%) (Table S3).

#### Narrative synthesis

Gong et al. (2020) [41] reported Tai Chi was superior in reducing depression scores on the *Zung* Self-Rating Depression Scale (SDS) (scale from 20 to 80; a smaller score indicates less depression), relative to usual pharmacological therapy in people with T2DM.

### Mood

Four studies [35, 58, 65, 67] reported the effects of Tai Chi on mood. We did not pool the data because of different comparisons and estimates (e.g., MD and median).

#### Narrative synthesis

Two studies [35, 58] assessed mood using the Symptom Checklist-90-Revised (SCL-90-R) by comparing Tai Chi in combination with usual care and usual care alone (including usual pharmacologic therapy, and health education). Barrow et al. (2007) [35] found no between-group differences in both SCL-90-R anxiety and depression scores in people with chronic heart failure, while Wang (2014) [58] found that Tai Chi plus usual care was superior to usual care alone in changing the SCL-90-R anxiety score in people with T2DM.

Another two studies [65, 67] measured changes in mood in respect to Tai Chi using the Profile of Mood States (POMS) scale in people with chronic heart failure. One study [65] involving 100 participants reported a significant improvement in median scores of total mood disturbance, depression, and vigour subscales of POMS in the Tai Chi group compared with the health education group, while the other study [67] involving 16 participants found no significant differences in POMS scores between the Tai Chi and aerobic exercise groups.

### Self-efficacy

#### Narrative synthesis

Two studies [52, 67] reported the effects of Tai Chi on self-efficacy. The Tai Chi and usual care group experienced a greater increase of self-efficacy than that of the usual care group in people with T2DM. Tai Chi was found to be equally effective in increasing self-efficacy scores compared with aerobic exercise in people with chronic heart failure.

### Quality of life

Thirty studies [35–40, 42–49, 51, 53, 54, 56, 57, 59–67, 70–72] reported the effects of Tai Chi on QoL.

#### Meta-analysis

Findings from the meta-analysis found that, compared with usual care alone, Tai Chi plus usual care significantly improved the total score of SF-36 (MD: 18.91, 95% CI: 12.80, 25.03, 3 studies, *I*^*2*^ = 54%) (Low certainty), mental health (MD: 7.86, 95% CI: 5.20, 10.52, 11 studies, *I*^*2*^ = 71%) (Low certainty), and bodily pain (MD: 6.76, 95% CI: 4.13, 9.39, 11 studies, *I*^*2*^ = 75%) (Low certainty) (Table 2, Table S5). The other domains of SF-36 all showed significant between-group differences in favour of the Tai Chi group, however, we did not use the pooled results due to considerable heterogeneity. Subgroup analyses could not explain all the heterogeneity, but found favourable effects of Tai Chi in improving: role limitation due to physical health for people with CVD risk factors (MD: 9.37, 95% CI: 6.33, 12.41, 6 studies, *I*^*2*^ = 15%); role limitation due to emotional health for people with CVD risk factors (MD: 8.04, 95% CI: 3.28, 12.81, 6 studies, *I*^*2*^ = 72%), and with CHD (MD: 16.09, 95% CI: 13.04, 19.14, 3 studies, *I*^*2*^ = 41%); energy/vitality in people with CVD risk factors (MD: 6.60, 95% CI: 3.23, 9.98, 6 studies, *I*^*2*^ = 57%); mental health in people with CVD risk factors (MD: 7.75, 95% CI: 3.77, 11.72, 6 studies, *I*^*2*^ = 69%), and with heart failure CVD risk factors (MD: 6.62, 95% CI: 1.04, 12.20, 2 studies, *I*^*2*^ = 55%); bodily pain in people with CVD risk factors (MD: 7.19, 95% CI: 3.23, 11.15, 6 studies, *I*^*2*^ = 59%), and with heart failure (MD: 5.92, 95% CI: 0.54, 11.30, 2 studies, *I*^*2*^ = 75%); and general health in people with CVD risk factors (MD: 9.95, 95% CI: 6.71, 13.18, 6 studies, *I*^*2*^ = 41%), and with heart failure (MD: 7.89, 95% CI: 2.72, 13.06, 2 studies, *I*^*2*^ = 70%) (Table S4).

Five studies [51, 64, 66, 71, 72] assessed QoL using the Minnesota Living with Heart Failure Questionnaire (MLHFQ), comparing Tai Chi in combination with usual care and the usual care alone in people with CHD and chronic heart failure. The pooled data found significantly favourable effects of Tai Chi; however, due to considerable heterogeneity which was not explained by subgroup analyses, we did not use the findings (Table S4). Another meta-analysis found that Tai Chi was equally effective in improving MLHFQ scores compared with that of aerobic exercise in people with chronic heart failure (MD: 1.55, 95% CI: − 8.50, 11.59, 2 studies, *I*^*2*^ = 0%) (Very low certainty) (Table 3, Table S5).

#### Narrative synthesis

The improvement of QoL measured by CQQC in the Tai Chi plus Chinese herbal medicine group was greater than that of the Chines herbal medicine alone group in people with CHD [70]. Similar findings were found in the Tai Chi combined with the usual care group, as measured by the abbreviated World Health Organization Quality of Life (WHOQOL-BREF) in people with hypertension [60]. Tai Chi had greater improvements in the mental component of SF-12 compared with the no treatment and aerobic exercise alone groups [37], and five domains of SF-36 compared with the aerobic exercise alone group [40]. When compared with non-exercise-based group activities, the Tai Chi group experienced greater improvement in six domains of SF-12, including role limitation due to physical health, role limitation due to emotional health, energy/vitality, mental health, social functioning, and bodily pain in people with hypertension [56]. Compared with usual care (i.e. usual stroke rehabilitation program including health education on stroke-specific symptom management via text messages), Song et al. (2021) [54] found that the Tai Chi group had greater increases in Stroke-Specific Quality of Life (SS-QOL) scores in the mood and thinking domains in people with stroke.

### Safety/adverse events

Eleven studies (11/37, 29.7%) reported safety/adverse events information. These studies involved people with T2DM, hypertension, CHD, chronic heart failure, and stroke.

#### Meta-analysis

Findings of the meta-analysis suggested that Tai Chi combined with usual care did not increase the risk of adverse events (RR: 0.50, 95% CI: 0.21, 1.20, 5 studies, *I*^*2*^ = 0%) (Very low certainty), compared with usual care alone (Table 2, Fig. S3). Subgroup analyses found consistent results (Table S4).

#### Narrative synthesis

No adverse events were reported to occur during Tai Chi sessions in eight studies. Three studies, involving people with CHD [47], stroke [54] and heart failure [67], reported no adverse events during their study periods, while other studies reported various adverse events in Tai Chi and control groups, including deaths (*n* = 9) [35, 42, 50, 65], heart failure decompensation (*n* = 1) [50], sepsis (*n* = 1) [50], fatigue (*n* = 3) [38, 50, 57], hospitalizations due to heart failure exacerbation, angina or shortness of breath (*n* = 11) [65, 66], arrhythmias (*n* = 2) [65], syncope (*n* = 2) [65], falls (*n* = 3) [65], dizziness [38], minor muscular soreness (*n* = 3) [38], worsening heart failure (*n* = 3) [35, 36] and worsening co-morbidities (*n* = 2) [35]. The authors concluded that these adverse events were unlikely to be caused by the Tai Chi interventions.

Tai Chi did not increase adverse events when compared with aerobic exercise [38], health education [65], or non-exercise-based group activities [57] (Table S3).

### Publication Bias

The funnel plot did not detect a publication bias in studies on Tai Chi for QoL in people with CVD or risk factors (Fig. S4).

## Discussion

This is a comprehensive systematic review evaluating the effects of Tai Chi on psychological well-being and QoL in people with CVD and/or cardiovascular risk factors. Tai Chi seems potentially effective in improving anxiety, depression, and QoL. The findings support the results of previous meta-analyses in the literature [27, 73].

Our findings related to SF-36 and MLHF improvements may bear clinical significance. Significant between-group differences in the mental health domain of SF-36 in favour of Tai Chi in combination with usual care, and the MD ranged from 6.76 in bodily pain and 7.86 in mental health to 14.18 points for role physical. Although there is still no consensus on the minimal important difference (MID) and minimal clinically important difference (MCID) of SF-36 in patients with cardiovascular risk factors or CVDs, prior systematic reviews of MID and MCID in health-related quality of life demonstrated that MID for SF-36 in patients with pulmonary fibrosis ranged from 2 to 4 points and in patients with prostate cancer ranged from 6 for mental health to 14 for role physical [74]. Accordingly, our findings related to Tai Chi for SF-36 may achieve clinical significance for patients. For MLHF, a meta-analysis of disease-specific health-related QoL questionnaires for heart failure demonstrated that interventions with small, expected effects, such as exercise programs, produced small score changes and interventions expected to produce large effects such as medications produced large score changes [75]. Another study exploring the minimal detectable change (MDC) and MCID of the MLHF found that the MDC ranged from 7.27 to 16.96 and the MCID related to “somewhat better” ranged from 3.59 to 19.14 points [76]. In our review, we found a significant difference in MLHF total scores in favour of Tai Chi in combination with usual care and the mean difference was 8.95 points, suggesting clinically significant findings.

There is still a lack of strong evidence that demonstrates unequivocally the beneficial effects of Tai Chi in decreasing stress, depression, anxiety, mood disturbance, and improving self-efficacy in people with CVD or cardiovascular risk factors. Although meta-analyses of a small number of studies demonstrated the favourable effects of Tai Chi on improving anxiety and depression, individual studies with various measurements also reported positive effects of Tai Chi in decreasing stress, anxiety, depression, and mood disturbance. More studies are needed to determine the effects of Tai Chi on psychological outcomes in this population.

Our meta-analysis indicates that Tai Chi appears to be safe to practice in people with CVD and/or cardiovascular risk factors. However, the overall safety of Tai Chi for people with CVD and/or cardiovascular risk factors remains unclear given that the majority of studies did not report safety information. It is worth noting that only less than 30% of the studies included in this review reported information on safety/adverse events. The low reporting rate of safety/adverse events is consistent with previous reviews. Wayne et al. (2014) [77] systematically assessed the frequency and quality of adverse events reported in English-language RCTs on Tai Chi and found that only 33% reported safety/adverse event information. Similarly, our previous reviews identified that only around 7.2 to 20% of clinical studies reported safety/adverse event information [28, 29]. Monitoring and reporting safety information in accordance with the CONSORT extension for reporting harms outcomes [78] are highly recommended for future research.

### Limitations and methodological challenges

This systematic review has several limitations. Firstly, the methodological quality of the included studies was generally poor. Less than half of the included studies reported the details of random number generation, allocation concealment, and blinding of outcome assessors. Although the corresponding authors were contacted to clarify the unclear or missing information in the papers, the response rate was very low. In addition, due to the nature of Tai Chi, blinding both participants and researchers to the interventions is not feasible, so the included studies might have a high risk of performance bias. We recommend future researchers blind outcome assessors in RCTs and report studies using the CONSORT (Consolidated Standards of Reporting Trials) 2010 statement [79] to improve research transparency.

Secondly, the results of individual studies may not achieve clinical significance in practice. For example, five included studies [41, 43, 55, 69, 72] used SDS (scale from 20 to 80) to assess depression. Previous studies with cardiovascular disease have often used a cut-off index score of 50 as a definition of clinical depression [80–82]. However, only three studies [41, 43, 72] had mean SDS scores above 50 in the Tai Chi and control groups at baseline.

Thirdly, most studies included in this review assessed the psychological outcomes and QoL as secondary outcomes. As a result, sample size calculations for these studies were based on physical biomarkers instead of psychological measures. Hence, the risk of underpowered evaluation may exist on the effects of psychological well-being and QoL in this cohort. It is uncertain whether a Tai Chi intervention that is primarily designed to assess psychological benefits may demonstrate a different finding.

Additionally, we utilised the SMD to compare the effects of Tai Chi across studies that used different scales to measure anxiety and depression, which increased the challenge to interpret the results clinically. However, we interpreted the SMD in the GRADE certainty rating using a general rule of thumb reported by Cohen [83], in which an SMD of 0.2 represents a small effect, 0.5 a medium effect, and 0.8 or larger a large effect. Since the instruments for anxiety and depression used in included studies might not be universally known for many clinicians and patients, SMD might be more interpretable than MD [84].

Finally, the small number of studies included in the meta-analyses for each outcome limit our confidence in the findings, particularly for anxiety and depression, which involved less than 10 studies in each meta-analysis. Also, we did not perform subgroup analyses to explore the effects of different Tai Chi styles and forms, because other styles and forms were derived directly or indirectly from *Chen* style and its forms, and the core principles and theories, such as balance, breathing, coordination, relaxation, and concentration are similar. Therefore, the findings of this study could not provide a specific recommendation based on Tai Chi styles/forms.

## Conclusion

Tai Chi is potentially effective in improving anxiety, depression, and quality of life, and seems to be safe to practice in people with CVD and/or cardiovascular risk factors. However, there is still a lack of strong evidence about the benefits of Tai Chi for the management of stress, self-efficacy, and mood disturbance in this population. The overall methodological quality of the included studies was poor. More high-quality RCTs exploring the beneficial effects of Tai Chi on psychological well-being and quality of life in this population are warranted.

## Supplementary Information

**Additional file 1: Table S1**. Search strategies. **Table S2.** Tai Chi interventions applied in the included studies. **Table S3.** Effect estimates of Tai Chi for psychological well-being and quality of life in people with or at risk of CVD. **Table S4.** Post-hoc subgroup analyses of Tai Chi for psychological well-being and quality of life in people with or at risk of CVD . **Table S5.** GRADE certainty assessment of the body of evidence. **Figure S1.** Risk of bias summary of included studies. **Figure S2.** Risk of bias graph of included studies**. Figure S3.** Forest plot of Tai Chi in combination with usual care on safety. **Figure S4.** Funnel plot of Tai Chi plus usual care versus usual care for mental health measured by SF-36.

## Data Availability

All data generated or analysed during this study are included in this published article and its supplementary information files.

## Abbreviations

CVD: Cardiovascular disease
CR: Cardiac rehabilitation
PROSPERO: International Prospective Register for Systematic Reviews
RCT: Randomised control trial
CENTRAL: Cochrane Central Register of Controlled Trials
CNKI: China National Knowledge Infrastructure
VJIP: VIP Journal Integration Platform
GRADE: Grading of Recommendations Assessment, Development and Evaluation
SoF: Summary of findings
MD: Mean difference
CI: Confidence interval
PRISMA: Preferred Reporting Items for Systematic Reviews and Meta-Analyses
ECG: Electrocardiogram
SF-12: 12-Item Short Form Health Survey
WHOQOL-100: World Health Organization Quality of Life
SF-36: Medical Outcomes Study 36-Item Short Form Health Survey
MacNew QLMI: MacNew Heart Disease Health-related Quality of Life Instrument
MLHFQ: Minnesota Living with Heart Failure Questionnaire
PSS-10: Perceived Stress Scale 10-item
STAI: State and Trait Anxiety Inventory
SAS: *Zung* Self-Rating Anxiety Scale
CES-D: Centre for Epidemiological Studies-Depression
SDS: *Zung* Self-Rating Depression Scale
POMS: Profile of Mood States
SCL-R: Symptom Checklist-90-Revised
