# Supplementary material for: Does tai chi improve psychological well-being and quality of life in patients with cardiovascular disease and/or cardiovascular risk factors? A systematic review
Source: BMC Complement Med Ther. 2022 Jan 4;22:3. doi: 10.1186/s12906-021-03482-0 (PMC8725570; doi:10.1186/s12906-021-03482-0)
Supplement: Supplementary file 1 — Additional file 1: Table S1. Search strategies. Table S2. Tai Chi interventions applied in the included studies. Table S3. Effect estimates of Tai Chi for psychological well-being and quality of life in people with or at risk of CVD. Table S4. Post-hoc subgroup analyses of Tai Chi for psychological well-being and quality of life in people with or at risk of CVD . Table S5. GRADE certainty assessment of the body of evidence. Figure S1. Risk of bias summary of included studies. Figure S2. Risk of bias graph of included studies. Figure S3. Forest plot of Tai Chi in combination with usual care on safety. Figure S4. Funnel plot of Tai Chi plus usual care versus usual care for mental health measured by SF-36. [file 12906_2021_3482_MOESM1_ESM.zip › Table S1 Search strategies_R3R4.docx]

**Table S1** Search strategies

| **Database** | **Number** | **Search items** |
| --- | --- | --- |
| PubMed | #1 | [Title/Abstract] (“Tai Chi” OR “Tai ji” OR “Ta’i chi” OR “taichi” OR “tai chi chuan” OR “taichi chuan” OR “taiji” OR “Tai Ji Quan” OR “taijiquan” OR “martial arts”) |
|  | #2 | [Title/Abstract] (“cardiovascular disease” OR “coronary heart disease” OR “stroke” OR “heart failure” OR “hypertension” OR “high blood pressure” OR “diabetes” OR “dyslipidaemia” OR “high cholesterol”) |
|  | #3 | [All fields] (“randomized controlled trial” OR “randomised controlled trial” OR “controlled clinical trial” OR “randomly” OR “clinical” OR “trial” OR “random” OR “randomised” OR “randomized”) |
|  | #4 | #1 and #2 and 3# |
| CNKI | #1 | [Abstract] (“*Tai_ji*” (Tai Chi) OR “*Tai_ji_quan*” (Tai Chi) |
|  | #2 | [Abstract] (“*Xin_xue_guan_bing*” (cardiovascular disease) OR “Guan_xin_bing” (coronary heart disease) OR “Zhong_feng” (stroke) OR “Nao_cu_zhong” (stroke) OR “Xin_shuai” (heart failure) OR “*Gao_xue_ya*” (hypertension) OR “*Tang*_*niao*_*bing*” (diabetes) OR “*Gao*_*xue*_*zhi*” (dyslipidaemia) |
|  | #3 | [All fields] (“*sui_ji*” (randomized or randomised)) |
|  | #4 | #1 and #2 and 3# |

**Note**: CNKI, China National Knowledge Infrastructure.
