# Supplementary material for: Does tai chi improve psychological well-being and quality of life in patients with cardiovascular disease and/or cardiovascular risk factors? A systematic review
Source: BMC Complement Med Ther. 2022 Jan 4;22:3. doi: 10.1186/s12906-021-03482-0 (PMC8725570; doi:10.1186/s12906-021-03482-0)
Supplement: Supplementary file 1 — Additional file 1: Table S1. Search strategies. Table S2. Tai Chi interventions applied in the included studies. Table S3. Effect estimates of Tai Chi for psychological well-being and quality of life in people with or at risk of CVD. Table S4. Post-hoc subgroup analyses of Tai Chi for psychological well-being and quality of life in people with or at risk of CVD . Table S5. GRADE certainty assessment of the body of evidence. Figure S1. Risk of bias summary of included studies. Figure S2. Risk of bias graph of included studies. Figure S3. Forest plot of Tai Chi in combination with usual care on safety. Figure S4. Funnel plot of Tai Chi plus usual care versus usual care for mental health measured by SF-36. [file 12906_2021_3482_MOESM1_ESM.zip › Table S2 Tai Chi interventions in included studies_R3R4.docx]

**Table S2** Tai Chi intervention applied in included studies

| **Study ID** | **Disease/**  **condition** | **Tai Chi intervention** | | | | |
| --- | --- | --- | --- | --- | --- | --- |
|  |  | **Style & Form** | **Instruction provider** | **Components** | **Timing (minutes/session; times/week)** | **Duration (weeks)** |
| Barrow DE 2007 | Symptomatic heart failure | Wu Chian Chuan style | A trained Tai Chi trainer, with a trained cardiac rehabilitation nurse also in attendance. | (1) Group class: Chi Kung (20 mins) for relaxation, the stillness of mind & mood; rest period (5 mins); Tai Chi (20 mins); cool down (5 mins). The practice was gradually increased, reaching full potential on week 8.  (2) Home practice encouraged. | 50 min/session; twice/week & home practice | 16 |
| Caminiti G 2011 | Chronic heart failure | A modified 10-form *Yang*-style | An experienced Tai Chi instructor | Group class: warm-up (10 mins), Tai Chi (30 mins), & cool down (10 mins). Week 1-8 learned the movements. Week 9-12 practiced the complete sequence, at the same speed. A trained cardiac rehabilitation therapist was also in attendance. | 50 min/session; three times/week | 12 |
| Chan AWK 2018 | Hypertension | 24 simplified *Yang*-style | A qualified and experienced Tai Chi master | (1) Group class: Tai Chi.  (2) Home practices were advised. | 60 min/session; twice/week & home practice 30 min/session at least five times per week | 12 |
| Cui H 2020 | Chronic heart disease | Modified Tai Chi | An experienced Tai Chi Master student from Beijing Sport University (National athlete Level A) | Group class: a warm-up exercise, three standing postures, and five movements, and a cool-down exercise. | 50 min; three times a week | 12 |
| Ding FM 2013 | Acute myocardial infarction after PCI | 42-form *Chen* style | NR | Group class: a warm-up exercise (15 mins), Tai Chi practice (30 mins), and cool-down exercise (15 mins). | 60 min/session; at least five times per week | 24 |
| Fan QY 2020 | Coronary heart disease | Tai Chi movement “Cloud hands’ | Nurse practitioner | Group class | NA | NA |
| Gong ZY 2020 | T2DM | Tai Chi (eight foundation movements and five steps) | NA | Group class: a warm-up exercise (10min), 50 min Tai Chi. | 60 min a day | 12 |
| Han QY 2010 | Hypertension | 24 simplified *Yang*-style | A professional Tai Chi instructor | (1) Before class: introduce the importance & methods of Tai Chi practice;  (2) Group class: warm-up (10-15 mins), Tai Chi (20-30 mins), and cool down (5 mins), focusing on the movements, breathing, and mind/consciousness. Target heart rate was monitored. | 45-60 min/session; 7-14 (1-2 sessions daily) times per week | 240 |
| Li Y 2019 | Chronic heart failure | five movements and 24-form *Yang* style | Tai Ch tutors | NA | 60 min a day; seven times per week | 24 |
| Liu J 2020 | Chronic heart failure | 24-form Tai Chi | NA | (1) Group class: According to the individual condition of the patient, the exercise intensity and the exercise volume should be adjusted according to the patient’s condition, and patient tolerance should be appropriate. | 50-60 min per day; 14 times per week | 40 |
| Luberto CM 2020 | Heart failure | 5 simplified *Yang*-style Tai Chi | 1 or 2 certified and experienced instructors (6 total study instructors with an average experience of 20 years) | (1) Group class: a warm-up exercise (i.e. weight shifting, arms swinging, gentle stretches, visualization techniques, and tractional breathing methods), focusing on releasing tension in the body, incorporating mindfulness and imagery into movement, increasing awareness of breathing, and promoting overall relaxation of body and mind; 5 simplified Tai Chi movements. Chairs were provided for resting, and patients could progress at their own pace.  (2) Home practice: videotape provided. | 60 min per time; 2 & >3 home practice/week | 12 |
| Ma CH 2018 | Hypertension | 24 simplified *Yang* style Tai Chi | Two professional trainers who were physical education teachers in a Sports University and had Tai Chi certification | 1. Group class: a warm-up exercise (10 mins), Tai Chi practice (70 mins), and cool-down exercises (10 mins). 2. Home practice was supported by Tai Chi video. | Twice 90-minute sessions weekly & encouraged the home practice | 29 |
| Ma CJ 2020 | Coronary heart disease | 24 simplified *Yang* style Tai Chi | Tai Chi instructor | Group class: a warm-up exercise (5-10 min), Tai Chi (40-50min), and cool-down exercise (5-10min) | 60 min per time; 3 times per week | 12 |
| Meng E 2014 | Type 2 diabetes | Tai Chi | NR | NR | NR | 12 |
| Pan XF 2016 | Chronic heart failure | 24 simplified *Yang*-style | NR | (1) Tai Chi practice in the morning. The intensity could be adjusted based on the target heart rate. Target heart rate = resting heart rate + resting heart rate x (60%-80%). ECG was provided to monitor patients' status;  (2) Home practice: heart rate monitored. | 30 min/session; seven times/week | 24 |
| Redwine LS 2019 | Heart failure | *Yang*-style Tai Chi Chuan-Short Form | A certified holistic health practitioner with > 10 yrs of experience teaching TC to chronically ill and older adults | (1) Group class: led by an experienced instructor, performed in a group, and provided at medical care or senior centres with minimal equipment and thus  easily disseminated. Descriptions of TC and RB class content are provided (SDC1). asked to exercise at a perceived exertion rating of “moderately difficult”, according to the Borg scale.  (2) Home practice: Written materials were provided to support home practice | 60 min; 2 & 10-20 min; 5/week home practice | 16 |
| Sang L 2015 | Chronic heart failure | Specially designed program | Health care providers in attendance | Group class: Tai Chi. Health care providers were also in attendance. | 15 min/session; seven times /week | 12 |
| Shen XY 2019 | Type 2 diabetes | 24 simplified *Yang*-style | Professional Tai Chi instructor | Trained 2 weeks before enrolling in the study | 60 min/session; 3 times/week | 12 |
| Shou XL 2019 | Hypertension | 24 simplified *Yang*-style | Professional rehabilitation therapists | 1. Participants were trained 2 weeks before entering into this study; 2. Group class: a warm-up exercise (10-15min), Tai Chi (20-30min), and a cool-down exercise. | 40-90 min/session; one to two times a day | 12 |
| Song R 2021 | Stroke | Modified Tai Chi | A trained Tai Chi instructor | 1. Group class: a 5-min warm-up, a 5-min Qigong, a 35-min of Tai Chi movements, and a 5-min cool-down. 2. Home practice: weekly follow-up call to remind home practice | 50 min; twice a week | 24 |
| Sun F 2014 | Hypertension | 24 simplified *Yang*-style | A professional Tai Chi instructor | Group class: practice in a comfortable and quiet environment, focusing on correct movements, breathing skills, and the mind/consciousness. | 120 min/session; seven times/week | 8 |
| Sun J 2015 | Hypertension | Tai Chi | An experienced trainer | (1) Group class: a variety of meditation techniques including breathing, balance, flexibility, concentration, calming, and stress-reduction techniques;  (2) Home practice | 90 min/session; twice/week and 120-minute home practice | 48 |
| Tsang T 2007 | Type 2 diabetes | Tai Chi for Diabetes (a 12-movement hybrid from Sun and *Yang* styles) | An investigator extensively trained | Group class: specific warm-up exercise for the whole body (10 mins); Tai Chi in its entirety (45 mins). Involving breathing techniques and visualization; cool-down exercises (5 mins). | 60 min/session; twice/week | 16 |
| Wang HP 2014 | Type 2 diabetes | 24 simplified *Yang*-style | NR | Group class: Patients could progress at their own pace and intensity. | 40 min/session; five times/week | 8 |
| Wang P 2009 | Type 2 diabetes | 24 simplified *Yang* style | A professional Tai Chi instructor | (1)   Before class: nurses and volunteers introduced the purpose, importance, and methods of Tai Chi practice;  (2)   Group class: Tai Chi program (45-60 mins), including a warm-up exercise, Tai Chi practice, and cool-down exercise, focusing on the movements, breathing, and mind or consciousness. The intensity could be adjusted based on the target heart rate. Target heart rate = resting heart rate + resting heart rate x (50%-70%). | 45-60 min/session; 5-7 times/week | 24 |
| Wang XB 2019 | Hypertension | 24 simplified *Yang*-style | Nurse practitioner | 1. In hospital, 2 weeks of the learning period. 2. Discharge of hospital, continue practice for 3 months, including a warm-up, Tai Chi, and cool-down exercise. | 30-45 min/time; 5 times per week | 8 |
| Wang XK 2013 | Acute myocardial infarction after PCI | 42-form *Chen* style | NR | Group class: Week 1-4 learned the sequence; Week 5-24 maintained practice: warm-up (15 mins), Tai Chi (30 mins), and cool down (15 mins). Heart rate, blood pressure & ECG were monitored before, during & after class. | 60 min/session; five times/week | 24 |
| Wang YH 2019 | Chronic heart failure | 24 simplified *Yang*-style | Physiologist | Group class with close supervision and safety monitor | 30-45 min/time, 5 times/week | 8 |
| Wu F 2010 | Type 2 diabetes | 24 simplified *Yang*-style | A physiologist with Tai Chi teaching experience | Group class: Practiced Tai Chi before breakfast till the heart rate reached 130-140 times /min. | 60 mins/session; at least three times/week | 24 |
| Yao CD 2010 | Chronic heart failure | 42-form *Chen* style | NR | Group class: Week 1-4 learned the complete sequence; Week 5-24 maintained practice: a complete set of Tai Chi (30 mins). The intensity could be adjusted based on the target heart rate. Target heart rate = resting heart rate + resting heart rate *(10%-20%). ECG telemeter was provided to monitor patients' status. | 5-15 min/session (30 mins per session after week 4); at least five times/week | 24 |
| Yeh GY 2004 | Chronic heart failure | 5 simplified *Yang*-style | An experienced Tai Chi instructor, with a physician also in attendance | (1)   Group class: warm-up (i.e. weight shifting, arms swinging, gentle stretches, visualization techniques, and traditional breathing), focusing on releasing tension in the physical body, incorporating mindfulness and imagery into movement, increasing awareness of breathing, and promoting overall relaxation of body & mind; 5-form simplified Tai Chi. Chairs were provided, and patients could progress at their own pace.  (2)   Home practice: videotape provided. | 60 min/session; twice/week and at least three times of home practice per week | 12 |
| Yeh GY 2011 | Chronic heart failure | 5 simplified *Yang*-style | 1 or 2 certified and experienced instructors (6 total study instructors with an average experience of 20 years) | (1)   Group class: a warm-up exercise (i.e. weight shifting, arms swinging, gentle stretches, visualization techniques, and tractional breathing methods), focusing on releasing tension in the body, incorporating mindfulness and imagery into movement, increasing awareness of breathing, and promoting overall relaxation of body and mind; 5 simplified Tai Chi movements. Chairs were provided for resting, and patients could progress at their own pace.  (2)   Home practice: videotape provided. | 60 min/session; twice/week & at least three times of home practice per week | 12 |
| Yeh GY 2013 | Heart failure with a preserved ejection fraction | 5-form simplified *Yang*-style | An experienced instructor | (1)   Group class: warm-up (i.e. weight shifting, arms swinging, gentle stretches, visualization techniques & traditional breathing), focusing on releasing tension in the physical body, incorporating mindfulness and imagery into movement, increasing awareness of breathing, and promoting overall relaxation of body & mind; 5-form simplified Tai Chi. Chairs were provided. Patients could progress at their own pace.  (2)   Home practice: videotape provided. | 60 min/session; twice/week and at least three times of home practice per week | 12 |
| Yin NN 2020 | Type 2 diabetes | 18-form *Chen*-style | Tai Chi instructor with >10 years of experience | Group class: a warm-up exercise, Tai Chi, and a cool-down exercise | 60 min/session; 5 times/week | 12 |
| Zhang EM 2014 | Type 2 diabetes with depression | 24 simplified *Yang*-style | NR | Group class: a warm-up exercise (5 mins); Tai Chi practice (50-60 mins) with background music under guidance in breathing, balance, and flexibility; cool-down exercises (5 mins). Week 1-2 learned the complete sequence; week 3-5 practiced single movements; week 6-8 practiced the complete sequence, focusing on the combination of mind, qi, and body; week 9-14 competitive practice. The intensity could be adjusted based on heart rate. The target heart rate = (220 -Age) x (65~85%) + resting heart rate. | 60 min/session; NR the times per week | 14 |
| Zhang GW 2020 | Coronary heart disease | Tai Chi | NR | Group class | 3 times/day; 30 min/time | 12 |
| Zhang SQ 2011 | Acute myocardial infarction after PCI | 42-form *Chen* style | NR | Group class: Week 1-4 learned the movements; Week 5-48 maintained practice: a complete set of Tai Chi (30 mins). Adjusted intensity based on the target heart rate. Target heart rate = resting heart rate + resting heart rate x (10%-20%). ECG telemeter was provided to monitor patients' status. | 5-15 min/session (30 mins per session after week 4); at least five times per week | 48 |
| Zhou B 2020 | Heart failure | 24-form *Yang*-style | NA | Group class: close supervision and safety monitor | 15 min/time, 5 times/week, after one month gradually increased to 20-40 min/time | 12 |

**Note**: NR, not reported; min, minute.
