# Supplementary material for: Does tai chi improve psychological well-being and quality of life in patients with cardiovascular disease and/or cardiovascular risk factors? A systematic review
Source: BMC Complement Med Ther. 2022 Jan 4;22:3. doi: 10.1186/s12906-021-03482-0 (PMC8725570; doi:10.1186/s12906-021-03482-0)
Supplement: Supplementary file 1 — Additional file 1: Table S1. Search strategies. Table S2. Tai Chi interventions applied in the included studies. Table S3. Effect estimates of Tai Chi for psychological well-being and quality of life in people with or at risk of CVD. Table S4. Post-hoc subgroup analyses of Tai Chi for psychological well-being and quality of life in people with or at risk of CVD . Table S5. GRADE certainty assessment of the body of evidence. Figure S1. Risk of bias summary of included studies. Figure S2. Risk of bias graph of included studies. Figure S3. Forest plot of Tai Chi in combination with usual care on safety. Figure S4. Funnel plot of Tai Chi plus usual care versus usual care for mental health measured by SF-36. [file 12906_2021_3482_MOESM1_ESM.zip › Table S3 Effect estimates_R3R4.docx]

**Table S3** Effect estimates of Tai Chi for psychological well-being and quality of life in people with or at risk of CVD

| **Outcomes and comparisons** | **Population** | **Effect estimate**  **MD/SMD/RR (95% CI), *I^2^*** | **No. of participants (studies)** | **Study ID** |
| --- | --- | --- | --- | --- |
| **Stress** |  |  |  |  |
| *Tai Chi versus No treatment* |  |  |  |  |
| Assessed with PSS-10 | HT | MD -2.95 [-5.17, -0.73] * | 131 (1 RCT) | Chan 2018 |
| *Tai Chi versus Aerobic exercise* |  |  |  |  |
| Assessed with PSS-10 | HT | MD -2.09 [-4.22, 0.04] | 131 (1 RCT) | Chan 2018 |
| *Tai Chi + Usual care versus Usual care* |  |  |  |  |
| Assessed with PSS-14 | CHD | MD -0.76 [-1.02, -0.50] * | 61 (1 RCT) | Liu 2020 |
| **Anxiety** |  |  |  |  |
| *Tai Chi + Usual care versus Usual care* |  |  |  |  |
| Assessed with SAS | CHD, HT | MD -14.28 [-19.80, -8.76], 92% ∆* | 349 (2 RCTs) | Li 2019, Wang XB 2019 |
| Assessed with HADS-A | CHD | MD -2.91 [-3.82, -2.00] * | 61 (1 RCT) | Liu 2020 |
| Pooled results of above studies | CHD, HT | SMD -2.13 [-2.55, -1.70], 60% ∆* | 410 (3 RCTs) | Li 2019, Wang XB 2019, Liu 2020 |
| *Tai Chi versus Health education* |  |  |  |  |
| Assessed with SAS | HT | MD -6.21 [-8.26, -4.16] * | 80 (1 RCT) | Sun F 2014 |
| *Tai Chi versus Usual care* |  |  |  |  |
| Assessed with SAS | T2DM | MD -8.50 [-13.80, -3.20] * | 20 (1 RCT) | Gong 2020 |
| **Depression** |  |  |  |  |
| *Tai Chi + Usual care versus Usual care* |  |  |  |  |
| Assessed with SDS | CHD, HF | MD -10.52 [-18.07, -2.96], 96% ∆* | 352 (2 RCTs) | Li 2019, Zhou 2020 |
| Assessed with HADS-D | CHD | MD -0.99 [-2.21, 0.23] | 61 (1 RCT) | Liu 2020 |
| Assessed with BDI | HF | MD -1.00 [-1.71, -0.29] * | 48 (1 RCT) | Redwine 2019 |
| Assessed with GDS | T2DM | MD -0.45 [-0.77, -0.13] * | 101 (1 RCT) | Shen 2019 |
| Assessed with CES-D | HT | MD -1.71 [-3.46, 0.04] * | 113 (1 RCT) | Ma CH 2018 |
| Pooled results of above studies | CHD, HF, T2DM | SMD -0.86 [-1.35, -0.37], 88% ∆* | 675 (6 RCTs) | Li 2019, Liu 2020, Ma CH 2018, Redwine 2019, Shen 2019, Zhou 2020 |
| *Tai Chi versus Aerobic exercise* |  |  |  |  |
| Assessed with SDS | T2DM | MD -0.15 [-2.75, 2.45] | 40 (1 RCT) | Zhang EM 2014 |
| Assessed with POMS | HF | MD -0.70 [-3.20, 1.80] | 16 (1 RCT) | Yeh 2013 |
| Pooled results of above studies | T2DM, HF | SMD -0.10 [-0.62, 0.43], 0%∆ | 56 (2 RCTs) | Yeh 2013, Zhang EM 2014 |
| *Tai Chi versus Health education* |  |  |  |  |
| Assessed with SDS | HT | MD -2.06 [-4.09, -0.03] * | 80 (1 RCT) | Sun F 2014 |
| Assessed with CES-D | T2DM | MD 0.83 [-0.78, 2.44] | 68 (1 RCT) | Yin 2020 |
| Pooled results of above studies | HT, T2DM | SMD -0.11 [-0.78, 0.56], 76% ∆ | 148 (2 RCTs) | Sun F 2014, Yin 2020 |
| *Tai Chi versus Usual care* |  |  |  |  |
| Assessed with SDS | T2DM | MD -8.20 [-11.88, -4.52] * | 20 (1 RCT) | Gong 2020 |
| **Mood** |  |  |  |  |
| *Tai Chi versus Aerobic exercise* |  |  |  |  |
| Assessed with POMS: |  |  |  |  |
| (1) POMS-Total mood disturbance | HF | MD 2.60 [-9.18, 14.38] | 16 (1 RCT) | Yeh 2013 |
| (2) POMS-Depression | HF | MD -0.70 [-3.20, 1.80] | 16 (1 RCT) | Yeh 2013 |
| *Tai Chi + Usual care versus Usual care* |  |  |  |  |
| Assessed with SCL-90: |  |  |  |  |
| (1) SCL-90-Anxiety | T2DM | MD -0.41 [-0.63, -0.19] * | 70 (1 RCT) | Wang HP 2014 |
| (2) SCL-90-Depression | T2DM | MD -0.02 [-0.25, 0.21] | 70 (1 RCT) | Wang HP 2014 |
| **Self-efficacy** |  |  |  |  |
| *Tai Chi + Usual care versus Usual care* |  |  |  |  |
| Assessed with SES6G | T2DM | MD 0.65 [0.46, 0.84] * | 101 (1 RCT) | Shen 2019 |
| *Tai Chi versus Aerobic exercise* |  |  |  |  |
| Assessed with SEBES | HF | MD 13.60 [-14.33, 41.53] | 16 (1 RCT) | Yeh 2013 |
| **Quality of life** |  |  |  |  |
| *Tai Chi + Chinese herbal medicine versus Chinese herbal medicine* |  |  |  |  |
| Assessed with CQQC | CHD | MD 17.28 [7.35, 27.21] * | 30 (1 RCT) | Zhang GW 2020 |
| *Tai Chi + Usual care versus Usual care* |  |  |  |  |
| Assessed with SF-36: | CHD, HT, 2DM, HF |  | 1124 (11 RCTs) | Han 2010, Li 2019, Liu 2020, Ma CH 2018, Ma CJ 2020, Meng 2014, Pan 2016, Shou 2019, Wang P 2009, Wang YH 2019, Wu 2010 |
| (1) Physical functioning |  | MD 7.81 [3.41, 12.22], 94% ∆* |  |  |
| (2) Role limitation due to physical health |  | MD 14.18 [6.78, 21.57], 97% ∆* |  |  |
| (3) Role limitation due to emotional health |  | MD 9.77 [5.74, 13.80], 87% ∆* |  |  |
| (4) Energy/Vitality |  | MD 10.84 [3.54, 18.14], 97% ∆* |  |  |
| (5) Mental health |  | MD 7.86 [5.20, 10.52], 71% ∆* |  |  |
| (6) Social functioning |  | MD 12.22 [4.65, 19.79], 97% ∆* |  |  |
| (7) Bodily pain |  | MD 6.76 [4.13, 9.39], 75% ∆* |  |  |
| (8) General health |  | MD 9.65 [6.91, 12.39], 77% ∆* |  |  |
| (9) Total score | CHD | MD 18.91 [12.80, 25.03], 54% ∆* | 369 (3 RCTs) | Ding 2013, Li 2019, Wang XK 2013 |
| Assessed with MLHFQ | HF, CHD | MD -8.95 [-11.84, -6.07], 93% ∆* | 515 (5 RCTs) | Sang 2015, Yao 2010, Yeh 2004, Zhang SQ 2011, Zhou 2020 |
| Assessed with WHOQOL-BREF | HT | MD 22.27 [18.92, 25.62] * | 100 (1 RCT) | Wang XB 2019 |
| *Tai Chi versus No treatment* |  |  |  |  |
| Assessed with SF-12: |  |  |  |  |
| (1) Physical Component Scale |  | MD 0.87 [-1.47, 3.21] | 131 (1 RCT) | Chan 2018 |
| (2) Mental Component Scale |  | MD 4.84 [1.78, 7.90] * | 131 (1 RCT) | Chan 2018 |
| *Tai Chi versus Aerobic exercise* |  |  |  |  |
| Assessed with MLHF | HF | MD 1.55 [-8.50, 11.59], 0% ∆ | 58 (2 RCTs) | Cui 2020, Yeh 2013 |
| Assessed with SF-12: |  |  |  |  |
| (1) Physical Component Scale | HT | MD 1.18 [-1.06, 3.42] | 131 (1 RCT) | Chan 2018 |
| (2) Mental Component Scale | HT | MD 3.39 [0.77, 6.01] * | 131 (1 RCT) | Chan 2018 |
| Assessed with SF-36: | CHD |  | 86 (1 RCT) | Fan 2020 |
| (1) Physical functioning |  | MD 10.04 [8.24, 11.84] * |  |  |
| (2) Role limitation due to physical health |  | MD 15.44 [13.98, 16.90] * |  |  |
| (3) Role limitation due to emotional health |  | MD 11.60 [9.87, 13.33] * |  |  |
| (4) Mental health |  | MD 22.66 [20.84, 24.48] * |  |  |
| (5) General health |  | MD 16.86 [14.81, 18.91] * |  |  |
| *Tai Chi versus Non-exercise-based group activities* |  |  |  |  |
| Assessed with SF-12: | HT |  | 266 (1 RCT) | Sun J 2015 |
| (1) Physical functioning |  | MD 3.36 [-0.61, 7.33] |  |  |
| (2) Role limitation due to physical health |  | MD 12.52 [8.62, 16.42] * |  |  |
| (3) Role limitation due to emotional health |  | MD 5.15 [0.81, 9.49] * |  |  |
| (4) Energy/Vitality |  | MD 9.33 [4.39, 14.27] * |  |  |
| (5) Mental health |  | MD 5.54 [1.02, 10.06] * |  |  |
| (6) Social functioning |  | MD 4.99 [0.80, 9.18] * |  |  |
| (7) Bodily pain |  | MD 14.04 [9.20, 18.88] * |  |  |
| (8) General health |  | MD -7.84 [-13.56, -2.12] |  |  |
| Assessed with SF-36: |  |  |  |  |
| Social functioning | T2DM | MD 4.00 [-15.14, 23.14] | 37 (1 RCT) | Tsang 2017 |
| *Tai Chi versus Usual care* |  |  |  |  |
| Assessed with SS-QOL: | Stroke |  | 34 (1 RCT) | Song 2021 |
| (1) Energy |  | MD 0.00 [-1.78, 1.78] |  |  |
| (2) Family roles |  | MD 0.34 [-1.83, 2.51] |  |  |
| (3) Language |  | MD 1.98 [-1.10, 5.06] |  |  |
| (4) Mobility |  | MD 3.34 [-0.55, 7.23] |  |  |
| (5) Mood |  | MD 3.09 [0.18, 6.00] * |  |  |
| (6) Social roles |  | MD 0.92 [-2.18, 4.02] |  |  |
| (7) Personality |  | MD 0.79 [-1.44, 3.02] |  |  |
| (8) Thinking |  | MD 2.27 [0.46, 4.08] * |  |  |
| (9) Self-care |  | MD 2.78 [-0.18, 5.74] |  |  |
| **Safety** |  |  |  |  |
| *Tai Chi + Usual care versus Usual care* | HF, HT | RR 0.50 [0.21, 1.20], 0% ∆ | 248 (5 RCTs) | Barrow 2007, Caminiti 2011, Han QY 2010, Redwine 2019, Yeh 2004 |
| *Tai Chi versus Aerobic exercise* | HF | RR 1.21 [0.31, 4.77] | 42 (1 RCT) | Cui H 2020 |
| *Tai Chi versus Health education* | HF | RR 0.55 [0.18, 1.64] | 100 (1 RCT) | Yeh GY 2011 |
| *Tai Chi versus Non-exercise-based group activities* | T2DM | RR 3.32 [0.14, 76.60] | 38 (1 RCT) | Tsang T 2007 |

**Abbreviations:** CI, confidence interval; *, the effect estimate favours Tai Chi group; ∆, result from Meta-analysis. PSS-10, Perceived Stress Scale 10-item; CES-D, Centre for Epidemiological Studies-Depression; SDS, Zung Self-Rating Depression Sale; STAI, State and Trait Anxiety Inventory; SAS, Zung Self-Rating Anxiety Scale; POMS, Profile of Mood States; SCL-90, Symptom Checklist-90-Revised; SEBES, Self-Efficacy-Barriers to Exercise Scale; SES6G, Self-Efficacy for Managing Chronic Disease 6-Item Scale; CQQC, China Questionnaire of Quality of Life in Patients with Cardiovascular Disease; WHOQOL-BREF, abbreviated World Health Organization Quality of Life; MLHFQ, Minnesota Living with Heart Failure Questionnaire; SF-12, 12-Item Short Form Health Survey; SF-36, 36-Item Short Form Health Survey; SS-QOL, Stroke-Specific Quality of Life questionnaire. HT, hypertension; CHD, coronary heart disease; HF, chronic heart failure; T2DM, type 2 diabetes.
