# Supplementary material for: Does tai chi improve psychological well-being and quality of life in patients with cardiovascular disease and/or cardiovascular risk factors? A systematic review
Source: BMC Complement Med Ther. 2022 Jan 4;22:3. doi: 10.1186/s12906-021-03482-0 (PMC8725570; doi:10.1186/s12906-021-03482-0)
Supplement: Supplementary file 1 — Additional file 1: Table S1. Search strategies. Table S2. Tai Chi interventions applied in the included studies. Table S3. Effect estimates of Tai Chi for psychological well-being and quality of life in people with or at risk of CVD. Table S4. Post-hoc subgroup analyses of Tai Chi for psychological well-being and quality of life in people with or at risk of CVD . Table S5. GRADE certainty assessment of the body of evidence. Figure S1. Risk of bias summary of included studies. Figure S2. Risk of bias graph of included studies. Figure S3. Forest plot of Tai Chi in combination with usual care on safety. Figure S4. Funnel plot of Tai Chi plus usual care versus usual care for mental health measured by SF-36. [file 12906_2021_3482_MOESM1_ESM.zip › Table S4 Post-hoc subgroup analyses_effect estimates_R3R4.docx]

**Table S4** Post-hoc subgroup analyses of Tai Chi for psychological well-being and quality of life in people with or at risk of CVD

| **Outcomes and comparisons** | **Effect estimate**  **MD/SMD/RR (95% CI), *I^2^*** | **No. of participants (studies)** | **Study ID** |
| --- | --- | --- | --- |
| **Anxiety** |  |  |  |
| *Tai Chi + Usual care versus Usual care* |  |  |  |
| Meta-analysis results for CHD and HT | SMD -2.13 [-2.92, -1.88], 60% ∆* | 410 (3 RCTs) | Li 2019, Liu 2020, Wang XB 2019 |
| Subgroup: CHD |  |  |  |
| Pooled results | SMD -1.98 [-2.65, -1.31], 76% ∆* | 310 (2 RCTs) | Li 2019, Liu 2020 |
| Subgroup: HT |  |  |  |
| Assessed with SAS | MD -17.24 [-20.04, -14.44] * | 100 (1 RCT) | Wang XB 2019 |
| **Depression** |  |  |  |
| *Tai Chi + Usual care versus Usual care* |  |  |  |
| Meta-analysis results for CHD, HF, T2DM | SMD -0.86 [-1.35, -0.37], 88% ∆* | 675 (6 RCTs) | Li 2019, Liu 2020, Ma CH 2018, Redwine 2019, Shen 2019, Zhou 2020 |
| Subgroup: CHD |  |  |  |
| Pooled results | SMD -1.02 [-1.85, -0.98], 94% ∆* | 310 (2 RCTs) | Li 2019, Liu 2020 |
| Subgroup: HF |  |  |  |
| Pooled results | SMD -0.85 [-1.52, -0.17], 85% ∆* | 264 (3 RCTs) | Ma CH 2018, Redwine 2019, Zhou 2020 |
| Subgroup: T2DM |  |  |  |
| Assessed with GDS | MD -0.45 [-0.77, -0.13] * | 101 (1 RCT) | Shen 2019 |
| *Tai Chi versus Aerobic exercise* |  |  |  |
| Meta-analysis results for T2DM and HF | SMD -0.10 [-0.62, 0.43], 0%∆ | 56 (2 RCTs) | Yeh 2013, Zhang EM 2014 |
| Subgroup: T2DM | MD -0.15 [-2.75, 2.45] | 40 (1 RCT) | Zhang EM 2014 |
| Assessed with SDS |  |  |  |
| Subgroup: HF |  |  |  |
| Assessed with POMS | MD -0.70 [-3.20, 1.80] | 16 (1 RCT) | Yeh 2013 |
| **Quality of life** |  |  |  |
| *Tai Chi + Usual care versus Usual care* |  |  |  |
| Meta-analysis of QoL assessed with SF-36: |  | 1124 (11 RCTs) | Han 2010, Li 2019, Liu 2020, Ma CH 2018, Ma CJ 2020, Meng 2014, Pan 2016, Shou 2019, Wang P 2009, Wang YH 2019, Wu 2010 |
| (1) Physical functioning | MD 7.81 [3.41, 12.22], 94% ∆* | 1124 (11 RCTs) | Han 2010, Li 2019, Liu 2020, Ma CH 2018, Ma CJ 2020, Meng 2014, Pan 2016, Shou 2019, Wang P 2009, Wang YH 2019, Wu 2010 |
| Subgroup: T2DM & HT | MD 5.38 [1.51, 9.25], 84% ∆* | 673 (6 RCTs) | Han 2010, Ma CH 2018, Meng 2014, Shou 2019, Wang P 2009, Wu 2010 |
| Subgroup: CHD | MD 14.19 [8.03, 20.35], 88% ∆* | 340 (3 RCTs) | Li 2019, Liu 2020, Ma CJ 2020 |
| Subgroup: HF | MD 5.38 [-0.89, 11.64], 75% ∆ | 111 (2 RCTs) | Pan 2016, Wang YH 2019 |
| (2) Role limitation due to physical health | MD 14.18 [6.78, 21.57], 97% ∆* | 1124 (11 RCTs) | Han 2010, Li 2019, Liu 2020, Ma CH 2018, Ma CJ 2020, Meng 2014, Pan 2016, Shou 2019, Wang P 2009, Wang YH 2019, Wu 2010 |
| Subgroup: T2DM & HT | MD 9.37 [6.33, 12.41], 15% ∆* | 673 (6 RCTs) | Han 2010, Ma CH 2018, Meng 2014, Shou 2019, Wang P 2009, Wu 2010 |
| Subgroup: CHD | MD 25.45 [13.61, 37.29], 96% ∆* | 340 (3 RCTs) | Li 2019, Liu 2020, Ma CJ 2020 |
| Subgroup: HF | MD 11.40 [4.65, 18.16], 77% ∆* | 111 (2 RCTs) | Pan 2016, Wang YH 2019 |
| (3) Role limitation due to emotional health | MD 9.77 [5.74, 13.80], 87% ∆* | 1124 (11 RCTs) | Han 2010, Li 2019, Liu 2020, Ma CH 2018, Ma CJ 2020, Meng 2014, Pan 2016, Shou 2019, Wang P 2009, Wang YH 2019, Wu 2010 |
| Subgroup: T2DM & HT | MD 8.04 [3.28, 12.81], 72% ∆* | 673 (6 RCTs) | Han 2010, Ma CH 2018, Meng 2014, Shou 2019, Wang P 2009, Wu 2010 |
| Subgroup: CHD | MD 16.09 [13.04, 19.14], 41% ∆* | 340 (3 RCTs) | Li 2019, Liu 2020, Ma CJ 2020 |
| Subgroup: HF | MD 5.00 [-1.46, 11.47], 73% ∆ | 111 (2 RCTs) | Pan 2016, Wang YH 2019 |
| (4) Energy/Vitality | MD 10.84 [3.54, 18.14], 97% ∆* | 1124 (11 RCTs) | Han 2010, Li 2019, Liu 2020, Ma CH 2018, Ma CJ 2020, Meng 2014, Pan 2016, Shou 2019, Wang P 2009, Wang YH 2019, Wu 2010 |
| Subgroup: T2DM & HT | MD 6.60 [3.23, 9.98], 57% ∆* | 673 (6 RCTs) | Han 2010, Ma CH 2018, Meng 2014, Shou 2019, Wang P 2009, Wu 2010 |
| Subgroup: CHD | MD 21.50 [11.03, 31.97], 95% ∆* | 340 (3 RCTs) | Li 2019, Liu 2020, Ma CJ 2020 |
| Subgroup: HF | MD 8.37 [0.45, 16.28], 85% ∆* | 111 (2 RCTs) | Pan 2016, Wang YH 2019 |
| (5) Mental health | MD 7.86 [5.20, 10.52], 71% ∆* | 1124 (11 RCTs) | Han 2010, Li 2019, Liu 2020, Ma CH 2018, Ma CJ 2020, Meng 2014, Pan 2016, Shou 2019, Wang P 2009, Wang YH 2019, Wu 2010 |
| Subgroup: T2DM & HT | MD 7.75 [3.77, 11.72], 69% ∆* | 673 (6 RCTs) | Han 2010, Ma CH 2018, Meng 2014, Shou 2019, Wang P 2009, Wu 2010 |
| Subgroup: CHD | MD 8.60 [1.85, 15.35], 87% ∆* | 340 (3 RCTs) | Li 2019, Liu 2020, Ma CJ 2020 |
| Subgroup: HF | MD 6.62 [1.04, 12.20], 55% ∆* | 111 (2 RCTs) | Pan 2016, Wang YH 2019 |
| (6) Social functioning | MD 12.22 [4.65, 19.79], 97% ∆* | 1124 (11 RCTs) | Han 2010, Li 2019, Liu 2020, Ma CH 2018, Ma CJ 2020, Meng 2014, Pan 2016, Shou 2019, Wang P 2009, Wang YH 2019, Wu 2010 |
| Subgroup: T2DM & HT | MD 10.23 [4.29, 16.17], 84% ∆* | 673 (6 RCTs) | Han 2010, Ma CH 2018, Meng 2014, Shou 2019, Wang P 2009, Wu 2010 |
| Subgroup: CHD | MD 21.68 [7.81, 35.56], 97% ∆* | 340 (3 RCTs) | Li 2019, Liu 2020, Ma CJ 2020 |
| Subgroup: HF | MD 4.91 [-0.77, 10.59], 67% ∆ | 111 (2 RCTs) | Pan 2016, Wang YH 2019 |
| (7) Bodily pain | MD 6.76 [4.13, 9.39], 75% ∆* | 1124 (11 RCTs) | Han 2010, Li 2019, Liu 2020, Ma CH 2018, Ma CJ 2020, Meng 2014, Pan 2016, Shou 2019, Wang P 2009, Wang YH 2019, Wu 2010 |
| Subgroup: T2DM & HT | MD 7.19 [3.23, 11.15], 59% ∆* | 673 (6 RCTs) | Han 2010, Ma CH 2018, Meng 2014, Shou 2019, Wang P 2009, Wu 2010 |
| Subgroup: CHD | MD 6.39 [-0.69, 13.46], 91% ∆* | 340 (3 RCTs) | Li 2019, Liu 2020, Ma CJ 2020 |
| Subgroup: HF | MD 5.92 [0.54, 11.30], 75% ∆* | 111 (2 RCTs) | Pan 2016, Wang YH 2019 |
| (8) General health | MD 9.65 [6.91, 12.39], 77% ∆* | 1124 (11 RCTs) | Han 2010, Li 2019, Liu 2020, Ma CH 2018, Ma CJ 2020, Meng 2014, Pan 2016, Shou 2019, Wang P 2009, Wang YH 2019, Wu 2010 |
| Subgroup: T2DM & HT | MD 9.95 [6.71, 13.18], 41% ∆* | 673 (6 RCTs) | Han 2010, Ma CH 2018, Meng 2014, Shou 2019, Wang P 2009, Wu 2010 |
| Subgroup: CHD | MD 11.17 [3.39, 18.96], 92% ∆* | 340 (3 RCTs) | Li 2019, Liu 2020, Ma CJ 2020 |
| Subgroup: HF | MD 7.89 [2.72, 13.06], 70% ∆* | 111 (2 RCTs) | Pan 2016, Wang YH 2019 |
| Meta-analysis of QoL assessed with MLHFQ for HF and CHD | MD -8.95 [-11.84, -6.07], 93% ∆* | 515 (5 RCTs) | Sang 2015, Yao 2010, Yeh 2004, Zhang SQ 2011, Zhou 2020 |
| Subgroup: CHD | MD -12.80 [-17.51, -8.09] * | 131 (1 RCT) | Zhang SQ 2011 |
| Subgroup: HF | MD -8.08 [-11.14, -5.03], 94% ∆* | 383 (4 RCTs) | Sang 2015, Yao 2010, Yeh 2004, Zhou 2020 |
| **Safety** |  |  |  |
| *Tai Chi + Usual care versus Usual care* | RR 0.50 [0.21, 1.20], 0% ∆ | 248 (5 RCTs) | Barrow 2007, Caminiti 2011, Han QY 2010, Redwine 2019, Yeh 2004 |
| Subgroup: HT | RR 0.19 [0.01, 3.73] | 58 (1 RCT) | Han 2010 |
| Subgroup: HF | RR 0.57 [0.03, 1.44], 0% ∆ | 190 (4 RCTs) | Barrow 2007, Caminiti 2011, Redwine 2019, Yeh 2004 |

**Abbreviations:** CI, confidence interval; *, the effect estimate favours Tai Chi group; ∆, result from Meta-analysis. PSS-10, Perceived Stress Scale 10-item; GDS, Geriatric Depression Scale; SDS, Zung Self-Rating Depression Sale; SAS, Zung Self-Rating Anxiety Scale; POMS, Profile of Mood States; SCL-90, Symptom Checklist-90-Revised; MLHFQ, Minnesota Living with Heart Failure Questionnaire; SF-12, 12-Item Short Form Health Survey; SF-36, 36-Item Short Form Health Survey. HT, hypertension; CHD, coronary heart disease; HF, chronic heart failure; T2DM, type 2 diabetes.
