# Supplementary material for: Does tai chi improve psychological well-being and quality of life in patients with cardiovascular disease and/or cardiovascular risk factors? A systematic review
Source: BMC Complement Med Ther. 2022 Jan 4;22:3. doi: 10.1186/s12906-021-03482-0 (PMC8725570; doi:10.1186/s12906-021-03482-0)
Supplement: Supplementary file 1 — Additional file 1: Table S1. Search strategies. Table S2. Tai Chi interventions applied in the included studies. Table S3. Effect estimates of Tai Chi for psychological well-being and quality of life in people with or at risk of CVD. Table S4. Post-hoc subgroup analyses of Tai Chi for psychological well-being and quality of life in people with or at risk of CVD . Table S5. GRADE certainty assessment of the body of evidence. Figure S1. Risk of bias summary of included studies. Figure S2. Risk of bias graph of included studies. Figure S3. Forest plot of Tai Chi in combination with usual care on safety. Figure S4. Funnel plot of Tai Chi plus usual care versus usual care for mental health measured by SF-36. [file 12906_2021_3482_MOESM1_ESM.zip › Table S5 GRADE certainty assessment of the evidence_R3R4.docx]

**Table S5** GRADE certainty assessment of the body of evidence

| **Outcomes** | **Estimate of effect  [95% CI]** | ***I^2^*** | **MCID** | **No. of participants (studies)** | **Risk of bias** | **Inconsistency** | **Imprecision** | **Indirectness** | **Publication bias** | **Visual certainty** | **GRADE Certainty*** |
| --- | --- | --- | --- | --- | --- | --- | --- | --- | --- | --- | --- |
| **Safety** |  |  |  |  |  |  |  |  |  |  |  |
| *Tai Chi + usual case vs Usual care* | RR 0.50 [0.21, 1.20] | 0% | n/a | 248 (5 RCTs) | S | NS | VS | NS | NS | ⨁◯◯◯ | VERY LOW |
| *Tai Chi vs aerobic exercise* | RR 1.21 [0.31, 4.77] | n/a | n/a | 42 (1 RCT) | S | NS | VS | NS | NS | ⨁◯◯◯ | VERY LOW |
| **Stress** |  |  |  |  |  |  |  |  |  |  |  |
| *Tai Chi + usual case vs Usual care* |  |  |  |  |  |  |  |  |  |  |  |
| Assessed with PSS-14 | MD -0.76 [-1.02, -0.50] | n/a | NI | 61 (1 RCT) | VS | NS | S | NS | NS | ⨁◯◯◯ | VERY LOW |
| *Tai Chi vs Aerobic exercise* |  |  |  |  |  |  |  |  |  |  |  |
| Assessed with PSS-10 | MD -2.09 [-4.22, 0.04] | n/a | 5 [1] | 131 (1 RCT) | NS | NS | VS | NS | NS | ⨁⨁◯◯ | LOW |
| **Anxiety** |  |  |  |  |  |  |  |  |  |  |  |
| *Tai Chi + usual case vs Usual care* |  |  |  |  |  |  |  |  |  |  |  |
| Pooled estimate | SMD -2.13 [-2.55, -1.70] | 60% | n/a | 410 (3 RCTs) | VS | NS | NS | NS | NS | ⨁⨁◯◯ | LOW |
| **Depression** |  |  |  |  |  |  |  |  |  |  |  |
| *Tai Chi + usual case vs Usual care* |  |  |  |  |  |  |  |  |  |  |  |
| Pooled results | SMD -0.86 [-1.35, -0.37] | 88% | n/a | 675 (6 RCTs) | S | S | NS | NS | NS | ⨁⨁◯◯ | LOW |
| *Tai Chi vs Aerobic exercise* |  |  |  |  |  |  |  |  |  |  |  |
| Pooled results | SMD -0.10 [-0.62, 0.43] | 0% | n/a | 56 (2 RCTs) | S | NS | VS | NS | NS | ⨁◯◯◯ | VERY LOW |
| **Quality of life** |  |  |  |  |  |  |  |  |  |  |  |
| *Tai Chi + usual care vs usual care* |  |  |  |  |  |  |  |  |  |  |  |
| Assessed with SF-36: Mental Health | MD 7.86 [5.20, 10.52] | 71% | 6 [2] | 1124 (11 RCTs) | VS | NS | NS | NS | NS^1^ | ⨁⨁◯◯ | LOW |
| Assessed with SF-36: Bodily Pain | MD 4.91 [-0.77, 10.59] | 67% | 25 [3] | 111 (2 RCTs) | VS | NS | VS | NS | NS | ⨁◯◯◯ | VERY LOW |
| Assessed with SF-36: Total score | MD 6.76 [4.13, 9.39] | 75% | 9 [2] | 1124 (11 RCTs) | VS | S | NS | NS | NS^1^ | ⨁◯◯◯ | VERY LOW |
| *Tai Chi vs Aerobic exercise* |  |  |  |  |  |  |  |  |  |  |  |
| Assessed by MLHF | MD 1.55 [-8.50, 11.59] | 0% | 5 to 7 [4] | 58 (2 RCTs) | S | NS | VS | NS | NS | ⨁◯◯◯ | VERY LOW |

**Abbreviations:** NS, not serious. S, serious. VS, very serious. 95%CI, 95% confidence interval; MCID, minimal clinically important difference; *I^2^*, the proportion of the variation in point estimates due to heterogeneity; MD, mean difference; SMD, standardised mean difference; RR, risk ratio; n/a, not applicable; NI, no information. PSS-10, Perceived Stress Scale 10-item; PSS-14, Perceived Stress Scale 14-item; MLHFQ, Minnesota Living with Heart Failure Questionnaire; SF-36, 36-Item Short Form Health Survey.

^1^ Symmetrical funnel plot.

*** GRADE RUBRIC**

**Risk of bias (RoB) [5]**

The key criteria for the RoB assessment include random number generation, allocation concealment, blinding of outcome assessor, incomplete accounting of patients and outcome events, selective outcome reporting bias, and other limitations.

a. RoB within a study: Low RoB: all key criteria were rated as low RoB; Moderate RoB: crucial limitation for one criterion or some limitations for multiple criteria sufficient to lower the confidence in the estimate of effect; High RoB: crucial limitation for one or more criteria sufficient to substantially lower the confidence in the estimate of effect.

b. RoB across studies: Low RoB: ≥ 75% studies were assessed as low RoB; High RoB: ≥ 75% studies were assessed as high RoB; otherwise, Moderate RoB.

Accordingly, the overall assessment of RoB across studies for each outcome is:

*Not serious*, do not downgrade: Low RoB; or Moderate RoB & stable effect estimate as indicated by sensitivity analysis of only low RoB of studies included.

*Serious*, rate down one level: Moderate RoB & no sensitivity analysis of only low RoB studies was conducted or if conducted, the effect estimates were unstable; or High RoB & the effect estimate is stable with sensitivity analysis when high RoB of studies excluded.

*Very serious*, rate down two levels: High RoB & no sensitivity analysis by excluding high RoB studies was conducted or if conducted, the effect estimates were unstable.

**Inconsistency [6]**

*Not serious*, do not rate down: heterogeneity *I^2^* ≤ 40%; or *I^2^* ≤ 75% & all studies favour one direction & confidence intervals (CIs) show widely overlapping (visual inspection); or *I^2^* ≤ 75% & subgroup/sensitivity analysis reduced the heterogeneity to less than 40% with stable effect estimate.

*Serious*, rate down one level: *I^2^* > 40% and does meet the criteria for *not serious*; or *I^2^* > 75% & all studies favour one direction (visual inspection); *I^2^* > 75% & subgroup/sensitivity analysis reduced the heterogeneity to less than 60% with stable effect estimate. (NOTE: if *I^2^* ≥ 90%, consider rate down two levels unless there is a strong case.)

*Very serious*, rate down two levels: *I^2^* > 75% and does not meet criteria for *serious* & point estimates vary widely across studies & CIs show minimal or no overlap.

**Indirectness [7]**

The quality of evidence for all outcomes was not rated down for indirectness, because the evidence comes from research that directly compares Tai Chi with controls in the populations in which we are interested and measures psychological wellbeing, quality of life and safety outcomes that are important to patients.

**Imprecision [8]**

**Optimum information size (OIS):** for SMD ≥ 400; for MD/HR based on sample size calculations for RCTs; relative/absolute risk OIS is met if > 4,000 participants & no. events > 100, alternatively, calculate OIS using RRRs of 20% to 30%, α of 0.05 and β of 0.2, or using Figs.4 and 5 in *GRADE guidelines 6. Rating the quality of evidence - imprecision* to determine OIS [8].

**Important benefit and harm included:** SMD ± 0.5; MD ± minimal clinically important difference (MCID); OR/RR/HR < 0.75 and > 1.25; ARR treatment outcomes and non-serious adverse events (AEs) ± 0.1; ARR serious AEs ± 0.01.

*Not serious*, does not rate down: OIS is met & 95% CI excludes no effect (i.e., RR of 1.0 or MD of 0); or OIS is met & 95% CI overlaps no effect & both important benefit and harm excluded.

*Serious*, rate down one level: OIS is not met & 95% CI excludes no effect; or OIS is met & 95% CI overlaps no effect but fails to exclude both important benefit and important harm; or OIS is not met & 95% CI overlaps no effect but fails to include both important benefit and harm.

*Very serious*, rate down two levels: OIS is not met & 95% CI overlaps no effect & both important benefit and harm included (i.e., very wide CI).

**Publication bias [9]**

*Not serious*, does not rate down: not assessable by the funnel plot, < 10 studies in the meta-analysis; or assessed as not present or probably not present.

*Serious*, rate down one level: assessed as ‘strongly suspected’ based on visual inspection of funnel plot and/or statistical test.

**References**:

1. Willert MV, Thulstrup AM, Hertz J: **Changes in stress and coping from a randomized controlled trial of a three-month stress management intervention**. *Scand J Work Environ Health* 2009, **35**(2):145-152.

2. Jayadevappa R, Cook R, Chhatre S: **Minimal important difference to infer changes in health-related quality of life-a systematic review**. *J Clin Epidemiol* 2017, **89**:188-198.

3. Wyrwich KW, Spertus JA, Kroenke K, Tierney WM, Babu AN, Wolinsky FD, Heart Disease Expert P: **Clinically important differences in health status for patients with heart disease: an expert consensus panel report**. *Am Heart J* 2004, **147**(4):615-622.

4. Mathai SC, Ghofrani HA, Mayer E, Pepke-Zaba J, Nikkho S, Simonneau G: **Quality of life in patients with chronic thromboembolic pulmonary hypertension**. *Eur Respir J* 2016, **48**(2):526-537.

5. Guyatt GH, Oxman AD, Vist G, Kunz R, Brozek J, Alonso-Coello P, Montori V, Akl EA, Djulbegovic B, Falck-Ytter Y *et al*: **GRADE guidelines: 4. Rating the quality of evidence--study limitations (risk of bias)**. *J Clin Epidemiol* 2011, **64**(4):407-415.

6. Guyatt GH, Oxman AD, Kunz R, Woodcock J, Brozek J, Helfand M, Alonso-Coello P, Glasziou P, Jaeschke R, Akl EA *et al*: **GRADE guidelines: 7. Rating the quality of evidence--inconsistency**. *J Clin Epidemiol* 2011, **64**(12):1294-1302.

7. Guyatt GH, Oxman AD, Kunz R, Woodcock J, Brozek J, Helfand M, Alonso-Coello P, Falck-Ytter Y, Jaeschke R, Vist G *et al*: **GRADE guidelines: 8. Rating the quality of evidence--indirectness**. *J Clin Epidemiol* 2011, **64**(12):1303-1310.

8. Guyatt GH, Oxman AD, Kunz R, Brozek J, Alonso-Coello P, Rind D, Devereaux PJ, Montori VM, Freyschuss B, Vist G *et al*: **GRADE guidelines: 6. Rating the quality of evidence--imprecision**. *J Clin Epidemiol* 2011, **64**(12):1283-1293.

9. Guyatt GH, Oxman AD, Montori V, Vist G, Kunz R, Brozek J, Alonso-Coello P, Djulbegovic B, Atkins D, Falck-Ytter Y *et al*: **GRADE guidelines: 5. Rating the quality of evidence--publication bias**. *J Clin Epidemiol* 2011, **64**(12):1277-1282.
