# Supplementary figures and images for: Does tai chi improve psychological well-being and quality of life in patients with cardiovascular disease and/or cardiovascular risk factors? A systematic review
Source: BMC Complement Med Ther. 2022 Jan 4;22:3. doi: 10.1186/s12906-021-03482-0 (PMC8725570; doi:10.1186/s12906-021-03482-0)

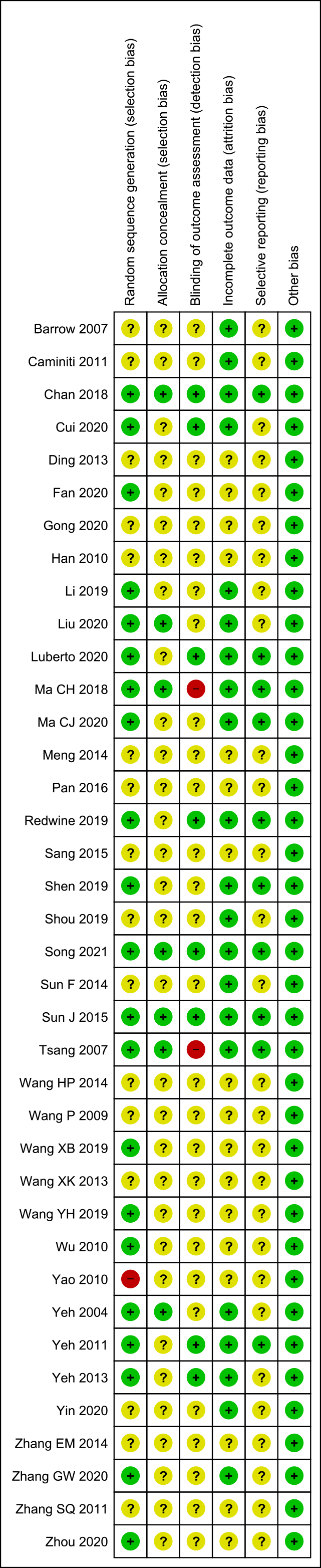

Supplement: Supplementary file 1 — Additional file 1: Table S1. Search strategies. Table S2. Tai Chi interventions applied in the included studies. Table S3. Effect estimates of Tai Chi for psychological well-being and quality of life in people with or at risk of CVD. Table S4. Post-hoc subgroup analyses of Tai Chi for psychological well-being and quality of life in people with or at risk of CVD . Table S5. GRADE certainty assessment of the body of evidence. Figure S1. Risk of bias summary of included studies. Figure S2. Risk of bias graph of included studies. Figure S3. Forest plot of Tai Chi in combination with usual care on safety. Figure S4. Funnel plot of Tai Chi plus usual care versus usual care for mental health measured by SF-36. [file 12906_2021_3482_MOESM1_ESM.zip › Figure S1_Risk of bias summary_R3R4.tif]

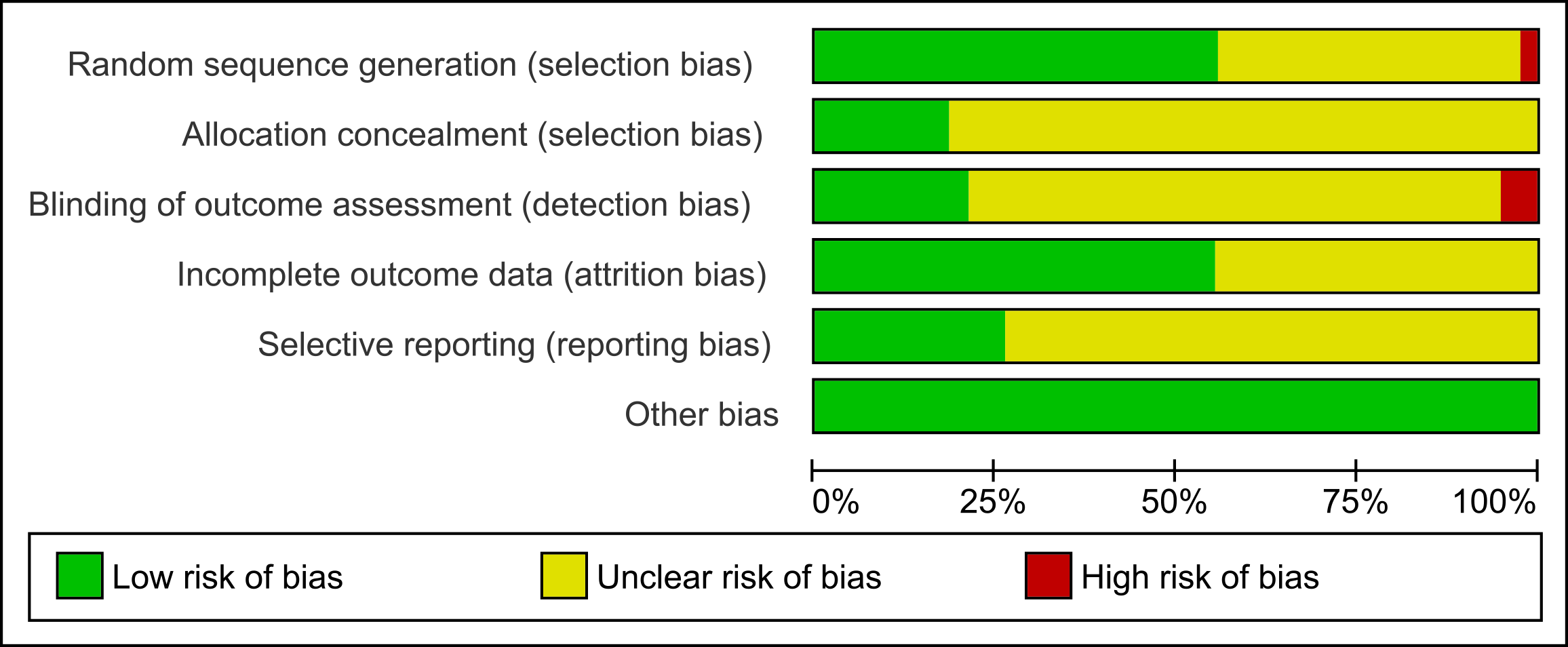

Supplement: Supplementary file 1 — Additional file 1: Table S1. Search strategies. Table S2. Tai Chi interventions applied in the included studies. Table S3. Effect estimates of Tai Chi for psychological well-being and quality of life in people with or at risk of CVD. Table S4. Post-hoc subgroup analyses of Tai Chi for psychological well-being and quality of life in people with or at risk of CVD . Table S5. GRADE certainty assessment of the body of evidence. Figure S1. Risk of bias summary of included studies. Figure S2. Risk of bias graph of included studies. Figure S3. Forest plot of Tai Chi in combination with usual care on safety. Figure S4. Funnel plot of Tai Chi plus usual care versus usual care for mental health measured by SF-36. [file 12906_2021_3482_MOESM1_ESM.zip › Figure S2_Risk of bias graph_R3R4.tif]

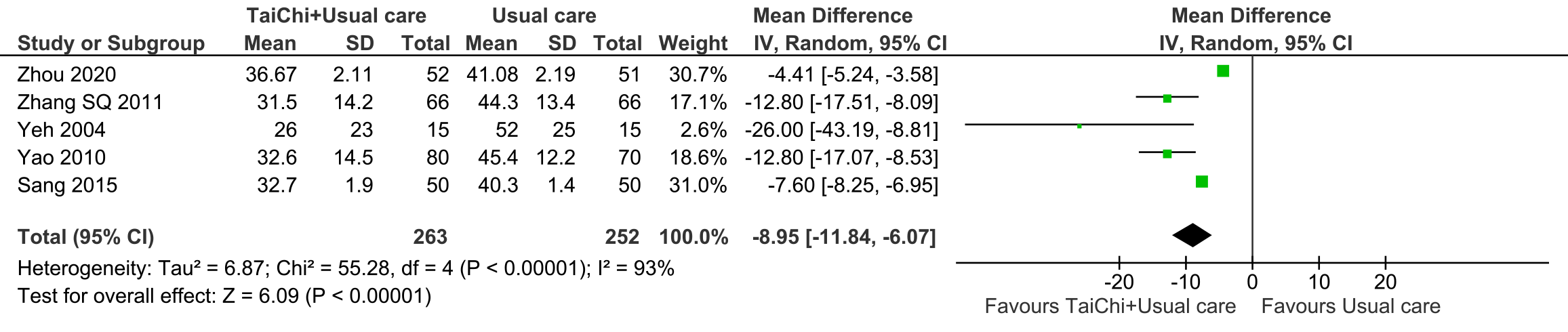

Supplement: Supplementary file 1 — Additional file 1: Table S1. Search strategies. Table S2. Tai Chi interventions applied in the included studies. Table S3. Effect estimates of Tai Chi for psychological well-being and quality of life in people with or at risk of CVD. Table S4. Post-hoc subgroup analyses of Tai Chi for psychological well-being and quality of life in people with or at risk of CVD . Table S5. GRADE certainty assessment of the body of evidence. Figure S1. Risk of bias summary of included studies. Figure S2. Risk of bias graph of included studies. Figure S3. Forest plot of Tai Chi in combination with usual care on safety. Figure S4. Funnel plot of Tai Chi plus usual care versus usual care for mental health measured by SF-36. [file 12906_2021_3482_MOESM1_ESM.zip › Figure S3_Forest plot of safety_R3R4.tif]

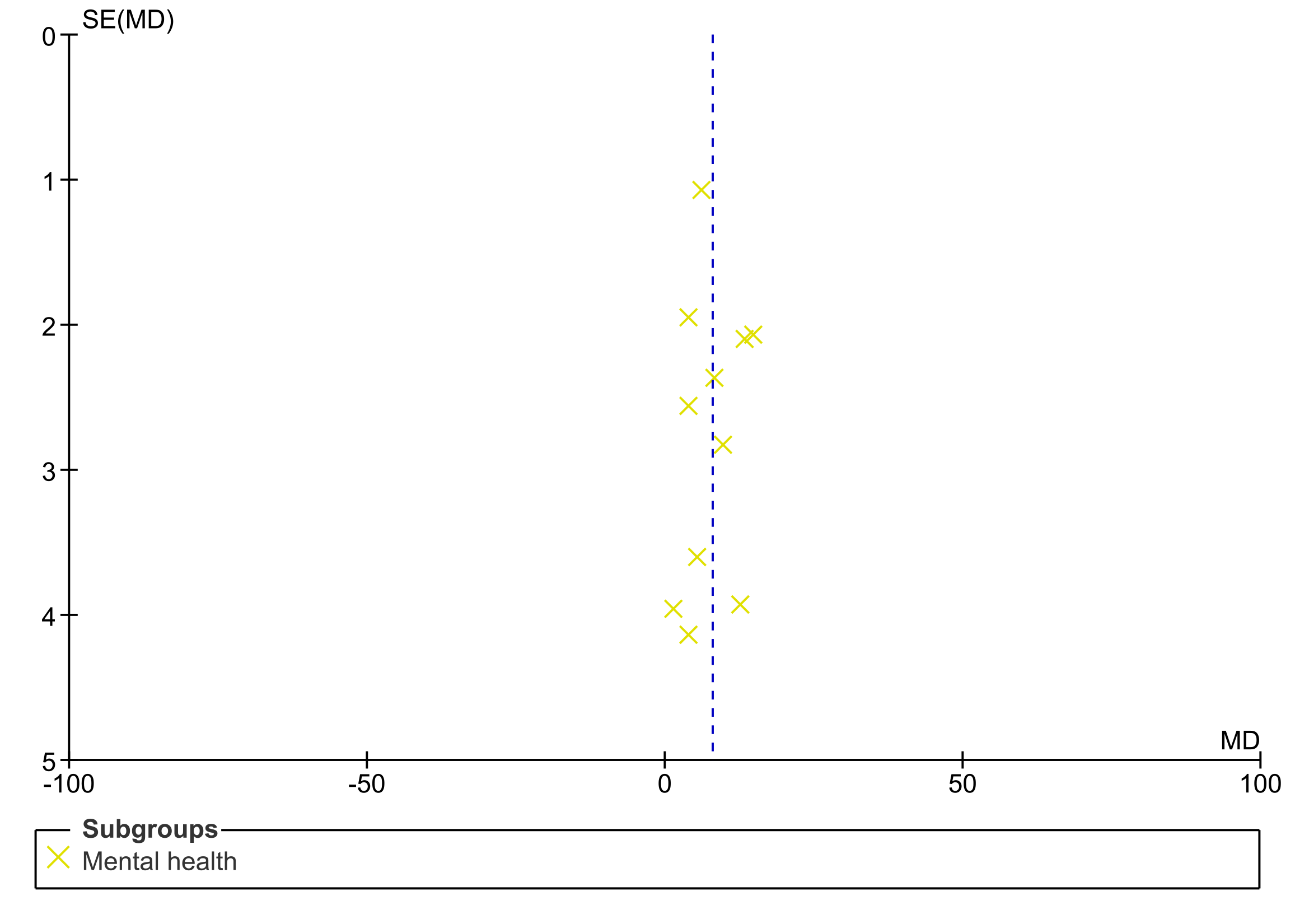

Supplement: Supplementary file 1 — Additional file 1: Table S1. Search strategies. Table S2. Tai Chi interventions applied in the included studies. Table S3. Effect estimates of Tai Chi for psychological well-being and quality of life in people with or at risk of CVD. Table S4. Post-hoc subgroup analyses of Tai Chi for psychological well-being and quality of life in people with or at risk of CVD . Table S5. GRADE certainty assessment of the body of evidence. Figure S1. Risk of bias summary of included studies. Figure S2. Risk of bias graph of included studies. Figure S3. Forest plot of Tai Chi in combination with usual care on safety. Figure S4. Funnel plot of Tai Chi plus usual care versus usual care for mental health measured by SF-36. [file 12906_2021_3482_MOESM1_ESM.zip › Figure S4_Funnel plot_R3R4.tif]
